# Prediction tools to prioritise hospitalised adult patients at risk of drug related problems: An umbrella review

**DOI:** 10.1371/journal.pdig.0001583

**Published:** 2026-08-06

**Authors:** Daria S. Gutteridge, Dona Babu, Jacquelina Stasinopoulos, Annabel Calder, Michael Bakker, Sally Marotti, Bronwin Patrickson, Karen Macolino, Craig Martin, Lijun Zhao, Niranjan Bidargaddi, Janet K. Sluggett

**Affiliations:** 1 Adelaide University, School of Allied Health and Human Performance, College of Health, Adelaide, Australia; 2 SA Pharmacy, SA Health, Adelaide, Australia; 3 College of Medicine and Public Health, Flinders University, Adelaide, Australia; 4 Adelaide University, School of Pharmacy and Biomedical Science, College of Health, Adelaide, Australia; 5 Digital Health Research Lab, College of Medicine and Public Health, Flinders University, Adelaide, Australia; 6 Commission on Excellence & Innovation in Health, SA Health, Adelaide, Australia; 7 South Australian Health and Medical Research Institute (SAHMRI), Adelaide, Australia; Lahore College for Women University, PAKISTAN

## Abstract

Risk prediction tools assist pharmacists to identify and prioritise hospitalised patients at risk of drug-related problems (DRPs) requiring clinical review. This umbrella review identifies existing risk prediction tools, summarises their performance, and highlights factors important for prioritising hospitalised adults at risk of DRPs for clinical review. A systematic search of three bibliographic databases from January 2010 to March 2024, identified systematic reviews that qualitatively or quantitatively examined patient- and/or medication-related factors in risk prediction or prioritisation models or tools for adult inpatients at risk of DRPs. Extracted data included citation, review type, number and date range of primary studies, population, setting, outcomes, and key risk factors. Risk prediction tools were summarised by country, sample size, performance metrics (discrimination, sensitivity, calibration), number of risk factors, and external validation. All extracted data was checked by a second reviewer. The standardized Joanna Briggs Institute critical appraisal instruments were used to assess the quality of eligible studies. Twenty systematic reviews met our inclusion criteria, of which 18 were of sufficient quality for synthesis. Selected articles covered 171 unique primary studies and 32 risk prediction tools, of which 12 demonstrated acceptable or good discrimination of which four also had good calibration. Two tools showed the highest potential for future clinical use based on their performance metrics and external validation data but would benefit from further validation in diverse populations. Commonly reported risk factors for DRPs were psycholeptic medications (reported in 13 systematic reviews), age, comorbidities and reduced renal function (n = 12 reviews). Based on the reviews included, no tools appear to be yet suitable for routine clinical practice due to limited external validation, calibration and unclear risk factor definitions; however, some show promise for further development and testing. Data on existing risk prediction tools and risk factors provides a foundation for refining or automating current models, for instance via machine learning approaches that can iteratively incorporate risk factors to ease use and improve prediction of DRPs within specific clinical settings.

## Author summary

Medications are essential for treating illness, but they can also be a source of harm, particularly in hospitals and during care transitions. Pharmacists can intervene to reduce risk but often have limited time and need better ways to identify who is most at risk of drug-related problems and requires closer clinical review. This study identified and evaluated existing research on tools designed to predict the risk of medication-related harm in hospitalised adults. We reviewed published summaries of previous studies to understand how these tools were developed, how well they performed, and which patient or medication-related factors were commonly applied. We found many different tools; however, based on the included reviews, none are currently ready for routine use in everyday clinical care. However, several showed promise, especially those that had been tested in more than one patient population. The commonly reported risk factors for drug-related problems were age, multiple medical conditions, reduced kidney function, and use of specific high-risk medications (i.e., psycholeptics). Our findings provide a strong foundation for improving existing tools or developing new automated approaches, including those using artificial intelligence (AI), to better support health professionals and improve patient safety in hospital settings.

## 1. Introduction

Drug related problems (DRPs) such as medication errors and adverse drug events (ADEs), which are adverse unintended consequences of medication use, are a common and major concern among hospitalised patients [1,2]. ADEs represent a major contributor to morbidity and mortality, with inpatient ADE rates ranging from 2.4% to 30% [3–13]. A previous systematic review of nine studies comprising >4,000 admissions found that 19% of inpatients experienced an ADE, of which one-third were considered preventable [14]. ADEs incur considerable health care costs, resulting in approximately $3.5 billion USD expenditure annually in the United States and £2.2 billion annually in the British National Health Service [13,15].

Clinical pharmacists play a critical role in minimising medication-related harm by detecting, resolving and preventing DRPs through clinical and pharmacovigilance activities, such as medication reconciliation and medication review [16]. Inpatient clinical pharmacy services are effective in reducing DRPs, with one study estimating a saving of approximately AUD $4.5 million annually in healthcare expenditure across eight major government-funded teaching hospitals in Australia [17]. However, comprehensive pharmacist services cannot be provided to all hospital inpatients due to workforce shortages (e.g., high ratios of patients to pharmacists), finite healthcare budgets, and increasing number and acuity of hospital admissions [2,18]. As a result, pharmacists must explicitly prioritise which patients receive medication reviews and other clinical pharmacy services. One study conducted in the UK reported that clinical pharmacists spend one hour daily prioritising patients and another half an hour gathering the clinical or laboratory data necessary for reconciling patients’ medication lists [19]. Hence, risk-prediction tools that assist healthcare professionals in streamlining medication reconciliation and targeting medication reviews to high-risk patients are a high priority. This is in line with international guideline recommendations for maximizing the value and impact of pharmacist services [20–22].

Risk prediction tools use patient and medication-related factors to estimate the risk of an adverse event such as ADE [23] to help identify and prioritise patients [19]. Directing health professionals to those most at risk can thereby reduce the risk of patient harm, improve efficiency in clinical practice, and limit avoidable hospital utilisation such as additional length of stay or readmission [19]. Although multiple risk prediction tools have been developed to identify patients at risk of DRPs, prior systematic reviews report that none have been implemented into routine clinical practice [24,25]. The growing availability of electronic medical record systems in hospitals and advances in artificial intelligence represent promising opportunities to implement automated patient prioritisation tools [26,27]. However, to support the development and implementation of effective digital health solutions, a comprehensive understanding of existing tools, including their performance, and commonly reported risk factors is required.

Multiple systematic reviews have examined risk factors associated with DRPs, however these typically focused on specific types of DRPs (e.g., medication errors, and ADEs [definitions of these terms can be found within the table in S1 Table]) or specific populations or settings (e.g., emergency departments [EDs]) [28–30]. Most existing reviews focus on estimating DRP prevalence, with risk factors often analysed as a secondary objective, with limited appraisal of prediction tool performance. Hence, there is a need to synthesize existing reviews to obtain a comprehensive overview of prediction tools and risk factors that are relevant across population groups and settings. The primary aim of this umbrella review is to identify available risk prediction tools and summarise their predictive performance, while the secondary aim is to narratively summarise key risk factors, including patient characteristics and medication-related factors, that are important for minimising DRPs in hospitalised patients.

## 2. Methods

This umbrella review adhered to the Preferred Reporting Items for Systematic Reviews and Meta-Analyses (PRISMA) guidelines and was conducted and reported in accordance with the Joanna Briggs Institute (JBI) Manual for Umbrella Reviews [31]. The protocol was registered on PROSPERO (CRD42024521195).

### 2.1. Search strategy

The following three databases were searched for academic literature on March 19, 2024: MEDLINE, Embase and CINAHL. The search strategy is available within the table in S2 Table. A combination of keywords and MeSH terms related to DRPs (e.g., adverse drug events) or medication safety in hospital settings, risk prediction models/tools, and systematic review were used. The search was restricted to English language and publications from January 2010 onwards, to maintain timeliness and relevance. No other filters were applied.

### 2.2. Eligibility criteria

Peer-reviewed systematic reviews that qualitatively or quantitatively examined patient characteristics (e.g., demographic, comorbidities) or medication-related factors in risk prediction or patient prioritisation models/tools to identify adult hospitalised patients at risk of DRPs (e.g., ADE, ME etc.) were included. Reviews were excluded if they focused primarily on paediatric populations (<18 years), outpatient services (including community care services and pre-operative services), discharge or transition of care services, mental health hospitals, a specific medication (as this is narrowly focused and does not reflect broader, system-level models of medication risks that consider multiple medications and patient factors simultaneously for prioritisation), or on DRPs that led to the hospital admission. Reviews that only discussed overall prediction models/tools without outlining individual patient or medication characteristics were excluded due to the second aim of this study. Consequently, some reviews that focused on AI, specifically machine learning prediction tools, were not eligible for inclusion because individual contributory risk factors were not detailed [26,27]. Conference abstracts, protocols, narrative/scoping reviews and dissertations were excluded.

### 2.3. Study selection

Identified references were extracted into Endnote, where duplicates were removed and subsequently uploaded into Covidence (Melbourne, 2023). Two reviewers (D.S.G. and A.C. or J.S.) independently identified potentially relevant studies based on abstract and titles. Potentially relevant full texts were independently screened by the same reviewers in Covidence and reasons for exclusion were recorded. Disagreements were resolved through discussion or if necessary, by the decision of a fourth reviewer (J.K.S).

### 2.4. Quality appraisal

Included studies were independently assessed for methodological quality by two reviewers, using the JBI standardized critical appraisal instruments for systematic reviews [32]; consistent with JBI umbrella review methodology, which includes published systematic reviews and meta-analyses as the analytical unit of the review [33]. The checklist comprises 11 items (see table in S3 Table), with each item answered as “Yes,” “No,” “Unclear,” or “NA”. The quality assessment focused on aspects related to risk factors and risk prediction models/tools, in line with our research question. Each “Yes” response was awarded one point. The percentage of “Yes” responses was calculated after excluding any NA items from the total, with reviews considered to be of satisfactory methodological quality if a score ≥70% was achieved [34]. Any disagreements between reviewers were resolved through discussion.

In addition, we extracted whether the systematic reviews applied a specific appraisal tool for prediction models, such as PROBAST (Prediction model Risk Of Bias ASsessment Tool), to assess risk of bias and applicability of the prediction models [35].

### 2.5. Data extraction

The JBI data extraction form for umbrella reviews was adapted to capture relevant information from the systematic reviews [36]. Extracted data included: first author, year of publication, date range of database search, type of review, number of primary studies included (i.e., original research) relevant to risk factors, population and setting, outcomes, and risk factors identified by the systematic review as being of particular importance across the included studies.

Risk prediction tools from the primary studies included in the systematic reviews were individually summarised. Extracted data included: first author and year, country of origin, study design, sample size, number of risk factors, and performance metrics for internal and external validation, where available. For a tool to be suitable for clinical implementation, adequate performance in both internal and external validation is required. In line with recommendations for reporting and appraising models (i.e., PROBAST [35]), we extracted measures of discrimination and calibration to assess model performance. Discrimination was assessed using the area under the receiver operating characteristic curve (AUROC) or concordance (C) statistic, which reflects the ability of a model to distinguishes between patients who will and will not experience the outcome of interest (e.g., ADE). We considered an AUROC of 0.70-0.80 to represent acceptable discrimination and >0.80 indicated good discrimination [25]. Sensitivity (i.e., the tool’s ability to correctly identify patients who will experience the outcome) and specificity (i.e., ability to correctly identify those who will not experience the outcome) were also extracted. Another key measure of predictive performance is a model’s calibration, which refers to the agreement between the predicted probability by a model and the actual outcomes. Perfect calibration is indicated by an intercept approaching 0 and a slope approaching 1, whereas a statistically significant Hosmer–Lemeshow test suggests poor model calibration [37].Details on external validation (i.e., testing the model in an independent dataset, often from a different patient population or setting, to assess how well it performs beyond the original cohort) were also extracted. Adequate power for developing multivariable prediction models was assessed using events per variable (EPV), with a minimum of 10 EPV generally recommended [38]. In line with the JBI Manual for Evidence Synthesis [39], data extraction was limited to the information presented by the systematic reviews. However, when conflicting data about the risk prediction tools were identified across systematic reviews, the original research article was consulted for verification. All data extracted was checked by at least one independent reviewer.

### 2.6. Data synthesis and study overlap

A qualitative approach was used to summarise risk factors and risk prediction tools identified by the systematic reviews. Overlaps in primary studies between systematic reviews were summarised using the corrected covered area (CCA) index (CCA = (N-r)/(r x c)-r; where N is the total number of primary studies (including duplicates); r is the number of unique primary studies; and c is the number of reviews) [40]. A CCA score of <5% was considered slight overlap, 5–10% indicated moderate overlap, and >10% represented high overlap [41]. However, as information on risk prediction tools was extracted individually for each tool, all identified systematic reviews were included regardless of overlap in primary studies.

Risk factors identified in the systematic review were narratively summarised and categorised into the following four categories: (1) patient characteristics, which refer to individual factors identified by the systematic review as being associated with DRPs (e.g., functional impairment, smoking status, demographics, and health service utilisation such as readmission/length of stay); (2) clinical characteristics (e.g., health conditions, laboratory findings); (3) medication classes, classified using the Anatomical Therapeutic Chemical (ATC) classification system [42]; and (4) other medication-related factors (i.e., characteristics of a medication regimen that are associated with DRPs such as polypharmacy, drug-drug interactions, potentially inappropriate medications or recent medication changes). Risk factor counts were based solely on the number of systematic reviews highlighting a given factor and should therefore be interpreted as a descriptive measure of reporting frequency rather than the relative importance, weighting, or effect size of the factor within individual prediction models. Furthermore, as overlap in primary studies was present across the included reviews, the reported frequencies may not represent fully independent lines of evidence.

## 3. Results

### 3.1. Study selection, quality and overlap

**Fig 1:**
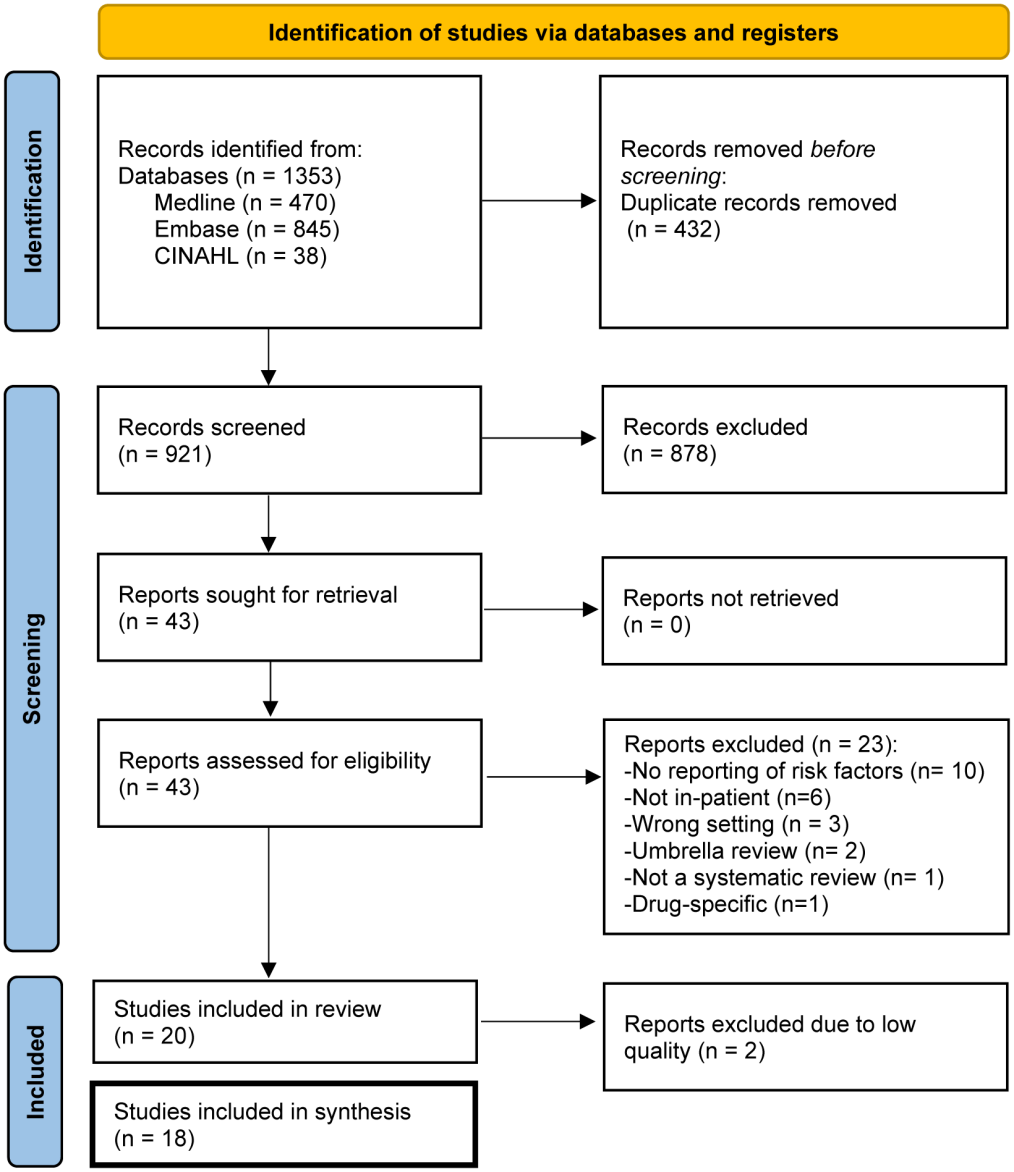
PRISMA Flow Diagram of study selection.

A total of 921 records were identified after exclusion of duplicate references, of which 43 articles were assessed for eligibility via full text (Fig 1). A total of 20 systematic reviews met our inclusion criteria and underwent quality appraisal (see table in S3 Table), of which 18 were of sufficient quality for data synthesis. Two reviews scoring 40% and 50% on the JBI appraisal respectively, were excluded [43,44] (see table in S3 Table). Notably, eight of the 18 studies included did not report methods to minimise errors in data extraction, such as having two individuals independently extract or check data [28,30,45–50]. Of the six reviews that specifically evaluated risk prediction tools four used a prediction-model specific risk-of bias tool, with two using CHARMS (Checklist for the critical Appraisal and data extraction for systematic Reviews of prediction Modelling Studies) [45,51], one using PROBAST [25], and one developed their own appraisal tool [52].

**Fig 2:**
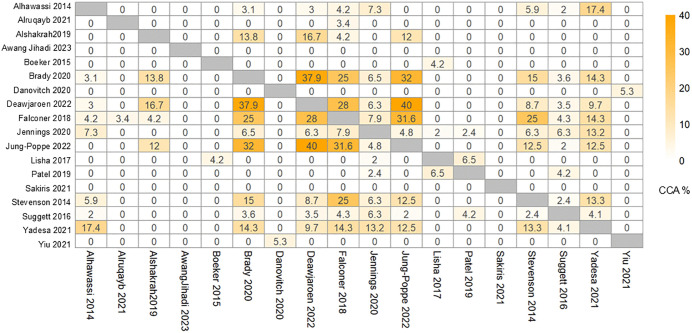
Pairwise corrected covered area (CCA) between systematic reviews (n = 18 reviews).

Fig 2 presents the pairwise overlap of primary studies across the 18 systematic reviews, as measured by the CCA. Pairwise overlap ranged from 0–40%, with high overlap (>10%) mainly observed among reviews focusing on risk prediction tools. The full list of the primary studies (n = 213) included in each systematic review can be found in the data in S1 Appendix. The total CCA across the 18 systematic reviews was low at 3.47%, with a total of 213 primary studies, of which 171 were unique.

### 3.2. Study characteristics

Table 1 summarises the characteristics of the 18 systematic reviews, which collectively reported data from 171 unique primary studies and 32 research tools [25,28–30,45–58]. Sixteen of the 18 reviews were published in the last 10 years, with the primary studies included in those reviews published between 1965 and 2023. The systematic reviews encompassed primary studies conducted across a diverse range of hospital settings, including ED, internal medicine, geriatric, surgical, cardiology, orthopaedic, intensive care, and stroke wards. Half of the systematic reviews (n = 9) focused on a single outcome (e.g. ADEs or adverse drug reactions (ADRs)) [28–30,47,50,53,56–58]. Nearly all the reviews (n = 17) employed a narrative synthesis of risk prediction tools and risk factors, while one review conducted a meta-analysis to examine patient characteristics and medication types associated with ADEs [30].

**Table 1:** Characteristics of the systematic reviews (n = 18) included in this umbrella review*.

Author, (year)	Aim of the systematic review	Date range of the review’s database search	Population/ hospital setting	No. of primary studies±	Outcomes	Risk prediction tools	Risk factors outlined^			
							Patient charac-teristics Y or N	Clinical characteristics Y or N	Medication classes Y or N	Other medication- related factors Y or N
Alhawassi et al. (2014) [28]	Assess the prevalence of ADRs before and during hospital admission in the older population and identify risk factors	2003-2013	Older population (>65 y)/ Acute care	14	ADR	No	Y (n = 6)	Y (n = 9)	Y (n = 5)	Y (n = 2)
Alruqayb et al. (2021) [29]	Investigate prevalence and risk factors of drug-related problems in hospitalised adults with chronic kidney disease (CKD)	Inception – 01/ 2020	Adults (>18 y) with CKD (stage I-IV)/ All departments	20	DRP	No	Y (n = 3)	Y (n = 1)	Y (n = 8)	Y (n = 2)
Alshakrah et al. (2019) [54]	Summarise patient prioritisation assessment tools for pharmaceutical care in hospitals	1990-2017	All in patients/All departments	19	Prioritise for pharmaceutical care (ADE, ADR, ME)	Yes	Y (n = 6)	Y (n = 3)	Y (n = 15)	Y (n = 7)
Awang Jihadi et al. (2023) [55]	Review and assess prevalence and risk factors of DRPs among hospitalised T2D patients	Inception- 06/2022	Adults (>18 y) with T2D/ General hospital ward	19	DRP, ADR, DDI, ME	No	Y (n = 2)	Y (n = 1)	Y (n = 4)	Y (n = 1)
Boeker et al. (2015) [30]	Identify patient characteristics and medications linked to (preventable) ADEs during admission in surgical and non-surgical patients	2000-April 2011	Adults/ Surgical & non-surgical departments	4	ADEs	No	Y (n = 1)	N	Y (n = 5)	Y (n = 1)
Brady et al. (2020) [45]	Identify and evaluate prediction tools to identify elderly patients at increased risk of DRPs or likely to need clinical pharmacist intervention	Inception – 05/2018	Older population (>65 y)/ All departments	19	ADRs, ADEs, ME, DRPs, hospital re-admissions, prescribing errors	Yes	Y (n = 6)	Y (n = 13)	Y (n = 9)	Y (n = 4)
Danovitch et al. (2020) [56]	Describe the rate of inpatient opioid overdose and associated risk factors, among medical and surgical patients admitted to hospital	Inception-July 2017	Adult/ Medical and surgical	4	Opioid-induced ADEs	No	Y (n = 3)	Y (n = 5)	Y (n = 2)	Y (n = 1)
Deawjaroen et al. (2022) [25]	Summarise risk prediction tools to identify hospitalised adults at risk of drug-related problems	Inception -December 2020	Adults (>18 y)/geriatric, cardiology, stroke ward, ICU.	21	DRP, ADE, ME, Prescribing errors, ADRs Medication harm	Yes	Y (n = 1)	Y (n = 2)	N	Y (n = 3)
Falconer et al. (2018) [51]	Critically appraise models developed for predicting adverse drug event risk in inpatients	Inception -December 2016	Adults/ surgical, medical, geriatric, stroke.	11	ADEs, unintentional discrepancies, prescribing errors, ME, ADR, DRP	Yes	Y (n = 3)	Y (n = 24)	Y (n = 9)	Y (n = 3)
Jennings et al. (2020) [57]	Determine the prevalence and clinical presentation of hospital-acquired ADRs in adults and identify associated medications	Inception- March 2020	Older population (>65 y)/critical care, geriatric, medical wards	30	ADRs	No	N	Y (n = 1)	Y (n = 21)	Y (n = 1)
Jung-Poppe et al. (2022) [46]	Identify risk factors that have been confirmed (or refuted) as suitable components for scoring tool to identify patients at risk of drug related problem	January 2011-August 2021	Adults (>18 y)/ surgical, geriatric, and internal medicine wards.	14	DRPS, ADRs, ADEs, ME	Yes	Y (n = 1)	Y (n = 2)	Y (n = 3)	Y (n = 1)
Lisha et al. (2017) [47]	Determine the prevalence, predictability, severity, determinants and medications implicated with ADRs in critical care settings	January 1995- June 2015	Adults and paediatrics/ Critical care units	21	ADR	No	Y (n = 1)	Y (n = 2)	Y (n = 7)	Y (n = 3)
Patel and Patel (2019) [58]	Determine the ADR following hospitalisation and explore the systems-organ class and drugs involved with fatal ADR	January 2000 – April 2018	Adult and Paediatric/ internal medicine, intensive care, neonatal ward		ADR	No	N	N	Y (n = 3)	Y (n = 1)
Sakiris et al. (2021) [48]	Determine the prevalence of ADEs at admission and during hospitalization in older inpatients with dementia. Identify commonly implicated drug classes and agents	Unclear	Older population with dementia	3	ADEs and ADRs	No	N	N	Y (n = 10)	Y (n = 1)
Stevenson et al. (2014) [52]	Identify and assess the quality of the validated ADR risk prediction model for adults over 65 years of age	Inception- November 2012	Older population (>65 y)	4	ADE, ADR	Yes	Y (n = 1)	Y (n = 6)	Y (n = 7)	Y (n = 2)
Suggett and Marriott, (2016) [49]	Identify the risk factors that are associated with medication related problems	1966 to July 2013	Adults and pre-adults (>16 y)/ General medical and surgical wards	38	ADE, ME, DRP, ADRs, Drug errors	No	Y (n = 3)	Y (n = 3)	Y (n = 10)	Y (n = 2)
Yadesa et al. (2021) [53]	Estimate the prevalence of ADRs in hospitalised older adults and identify the predictors across low- and high-income countries	Inception- December 2020	Older population (>60 y) acute care, stroke wards, and general ward.	13	ADRs	No	Y (n = 6)	Y (n = 9)	Y (n = 2)	Y (n = 3)
Yiu et al. (2021) [50]	Summarise risk factors and impacts of opioid-related adverse events in hospitalised patients.	Inception- April 2020	Adults/ Acute care (medical and surgical)	16	Opioid-related adverse drug events	No	Y (n = 2)	Y (n = 11)	Y (n = 2)	Y (n = 1)

*All systematic reviews used a narrative synthesis to identify risk factors, so a specific column was not used to describe the types of synthesis. The table in S4 Table provides detailed individual factors included within each category: Patient Characteristics, Clinical characteristics, medication classes, and other medication rug related factors.

ADE: adverse drug event, ADR: adverse drug reaction, DDI: drug-drug interactions, DRP: drug related problem, ICU: intensive care unit, MRP: medication related problem, ME: medication errors, T2D – Type 2 diabetes, Y: Yes, N: No

**^** No. of risk factors are based on the narrative text, i.e., the risk factors were highlighted by the systematic review as important in minimising drug related problems.

**±**Number of primary studies refers to the number of primary studies included in the systematic review that reported the risk factors and/or risk prediction tools relating to the adult population. Primary studies that only reported prevalence of an outcome or did not report risk factors or risk prediction tools were excluded.

### 3.3. Risk prediction tools

Six systematic reviews reported on risk prediction tools (see Table 2). Of the 32 tools identified, eight (25%) were developed for use in older inpatients (≥65 years) [59–66], while the remaining tools focused on the wider adult inpatient population (with and without inclusion of paediatric and obstetric populations) [67–91]. Risk prediction tools were developed and internally validated across a variety of clinical settings including internal medicine, surgical, rehabilitation, cardiology, and general medicine wards and EDs. The systematic reviews did not report whether data used to develop these tools were derived from electronic medication management systems or other digital health systems. None of the included tools described applying artificial intelligence to iteratively learn from an initial set of variables. The number of risk factors included in the tools ranged from 3 to 310 variables.

**Table 2:** Details of the risk prediction tools (n = 32) included in the systematic reviews*.

Author (year), Tool Name	Systematic reviews	Outcome	Country	Population/ Setting	Development/internal validation						External validation
					Design	Sample size/ Sufficient Power *	Discrimination AUROC [95% CI]	Sensitivity (SN)/ Specificity (SP)	Calibration	No. of risk factors	
Alassaad et al. (2015) [59], The 80 + score	Brady et al.	Time to re- hospitalisation, mortality	Sweden	Older population (≥80 y)/ Internal medicine	Prospective RCT	n= 400; (n=199 in intervention)/No	C-statistic 0.71	NR (P)	NR (SR)	7	NA
Audurier et al. (2021) [67], No name	DeawJaroen et al	MEs	France	Adult/ Internal medicine, surgical	Prospective cohort	Development: n = 1,256; Validation: n = 628/ NR (SR)	0.68(0.68- 0.73)	SN: 74% SF: 53%	NR (P)	4	NA
Bos et al. (2018) [68], No name	Jung-Poppe et al	Preventable ADE	Netherlands	Adult/ Surgical, urological, and orthopaedic wards	Prospective open intervention	Development: n = 6,780; Validation: bootstrap (200 samples)/ NR (SR)	NR (SR)	NR (SR)	NR (SR)	5	NA
Carlson and Phelps (2015) [69], No name	Alshakrah et al	Patients who benefit from medication reconciliation/monitoring	US	Paediatric and adult patients/ NR (SR)	Descriptive article	NA	NA	NA	NA	NR (SR)	NA
Cottrell et al. (2013) [70], No name	Alshakrah et al	Medication incidents	UK	Inpatients/ NR (SR)	Prospective cohort	n=15/No	NR (SR)	NR (SR)	NR (SR)	NR (SR)	NA
Covvey et al. (2015) [71], No name	Alshakrah et al	Pharmacist intervention opportunities, adverse events	UK	Obstetric patients/ NR (SR)	Retrospective chart review	n=175/ NR (SR)	NR (SR)	NR (SR)	NR (SR)	NR (SR)	NA
El Hajji et al. (2015) [72], No name	Alshakrah et al	Patients at high risk of admission and post-discharge mortality	UK	Inpatients with integrated medication manage-ment/ NR (SR)	Retrospective chart review	n=806/ NR (SR)	NR (SR)	NR (SR)	NR (SR)	NR (SR)	NA
Falconer et al. (2014) & (2017) [73,74], ART	Brady et al.; Deawjaroen et al.; Jung-Poppe et al.; Alshakrah et al.	ADEs	New Zealand	Adult/ Cardiology and general medicine	Prospective cohort	n = 235/ Yes	0.81 [0.72-0.89] (for top 4 factors)	NR (P)	NR (P)	25	NA
Falconer et al. (2021) [91], AIME model	Jung-Poppe et al; Deawjaroen et al.	Medication harm	Australia	Adult/ General medical, Geriatric assessment, rehabili-tation	Retrospective cohort	Development: n = 1,982; Validation: 200 resampling (bootstrap)/ NR (SR)	0.70 [0.65-0.74]	SN: 77% SF: 58%	Hosmer–Lemeshow’s test (P = 0.53)	10	NA
Geeson et al. (2019) [75], MOAT	Jung-Poppe et al; Deawjaroen et al.	Medication related problems	UK	Adult/ Medical wards, general emergency, geriatric medicine	Prospective cohort	n= 1,503, Validation: 200 resampling (bootstrap)/ NR (SR)	C-statistic = 0.66	SN: 90% between low-moderate SF: 30% low-moderate	Corrected slope = 0.86	11	NA
Hickson et al. (2017) [76], PAST	Brady et al.; Alshakrah et al.	Patient acuity level	UK	Adult/ Teaching Hospital	Quasi experimental service evaluation	n= 35/ NA	NR (P)	NR (P)	NR (P)	NR (P)	NA
Hohl et al. (2012) [77], ADE & ADR Clinical Decision Rules	Brady et al; Jung-Poppe et al	Primary: ADE, Secondary: ADR	Canada	Adult/ Emergency department	Prospective cohort	n= 1,591/ Unclear	NR (SR)	SN ADE: 97% [91.8-98.6]; SN ADR 91 [81.4-95.7] SF ADE 40% [37.7-42.9] SF ADR 59% [58.7-59.3]	NR (P)	9	Hohl et al. (2017) [92], (2018) [93]
Jeon et al. (2017) [78], No name	Alshakrah et al	Preventable ADE	US	Hospitalised patients/ NR (SR)	Systematic review and expert panel	n=37,391 (The American Society of Health System Pharmacists members)/ NA	NA	NA	NA	NR(SR)	NA
Kaufmann et al. (2018) [79], DART V2	Brady et al.; Deawjaroen et al.	DRPs	Switzer-land	Adult/ Orthopaedic, geriatric and internal medicine	Prospective cohort	Development: n = 164; Validation: n= 31/ NR (SR)	NR (P)	SN: 67%, SP: 88%	NR (P)	27	Staempfli et al. 2018 [94]
Lima et al. (2020) [80], No name	Jung-Poppe et al. 2022	ADRs	Brazil	Adult/ Neurology, mental health, nephrology, urology, cardiology, oncology, surgery, gastro-enterology, rheuma-tology	Retrospective Cohort	n = 1,029 (2/3 of cases and respective control for development, for validation 1/3 of sample)/ NR (SR)	NR (SR)	NR (SR)	NR (SR)	14	NA
Martinbiancho et al. (2011) [81], No name	Alshakrah et al	ADRs	Brazil	Inpatient adults and peadiatrics/ NR (SR)	Prospective observational	n=1,442/ NR (SR)	NR (SR)	NR (SR)	NR (SR)	NR (SR)	NA
McAuliffe et al. (2018) [82], MEDCOINS	Brady et al.	Readmission	US	Adult/ Hospital- all services	Retrospective cohort	n= 690/ Unclear	C-statistic 0.65[0.60-0.70]	NR (P)	Hosmer-Lemeshow goodness-of-fit = 0.99	12	NA
McElnay et al. (1997) [63], No name	Stevenson et al; Brady et al; Falconer et al	ADE, ADRs, adherence	Ireland	Older population (≥65 y)/ Medical surgical, general wards	Prospective cohort	Development: n = 929; Validation: n = 204/ Unclear	NR (SR)	SN: 41% SF: 69%	NR (P)	7	NA
Nguyen et al. (2017) [83], PRISMOR model	Jung-Poppe et al; Deawjaroen et al.; Falconer et al.; Alshakrah 2016	MEs	France	Adult/ Internal medicine, surgical	Prospective cohort	Development: n = 1,408; Validation: 500 samples (Bootstrap)/ Yes	C-statistics: 0.71	NR (P)	Corrected intercept = - 0.069 Slope = 0.926	11	NA
O’Mahony et al. (2018) [64], ADROP	Brady et al. Jung-Poppe et al.	ADRS	Ireland	Older population (≥65 y)/ General medicine, surgical, emergency,	Retrospective and prospective cohort	Development: n = 1,686; Validation: n = 530/ Unclear	Development: 0.62 [0.60-0.67]; validation:0.59 [0.53-0.65]	SN: NR (P) SF: NR (P)	NR (P)	8	NA
Onder et al. (2010) [60], GerontoNET ADR Risk Score	Brady et al.; Deawjaroen et al; Jung-Poppe et al; Stevenson et al; Falconer et al.	ADRs	Italy	Older population (≥65 y)/ Internal medicine	Retrospective cohort	n= 5,936/ Yes	0.71 [0.68-0.7]	SN: 68% SP: 65%	NR (SR)	6	Onder et al. (2010) [60]; O’Connor et al. (2012) [95]; Petrovic et al. (2017) [96]
Passarelli and Filho, (2007) [65], No name	Falconer et al.	ADRs	Brazil	Older population (≥ 60y)/Medical wards	Prospective cohort	n=186/ Unclear	Accuracy 70.4%	SN: 89% SP: 41%	Hosmer-Lemeshow p = 0.348	3	NA
Roten et al. (2010) [61], No name	Deawjaroen et al; Alshakrah et al.	DRPs	Switzerland	Geriatric/ Internal medicine	Prospective cohort	n= 501/NR (SR)	NR (P)	SN: 85% SF 60%	NR (P)	6	NA
Saedder et al. (2016) [86], MERIS	Brady et al.; Deawjaroen et al.; Jung-Poppe et al.; Alshakrah et al.	MEs	Denmark	Adult/Orthopaedic ward, internal medicine,	Retrospective and prospective cohort	Development: n = 249; Validation: n= 53/ Unclear	0.76, 0.87, 0.74, 0.66	SN: 64% SP: 75%	NR (P)	3	Bonnerup et al. 2016 [97]; Høj et al., 2021 [98]
Sakuma et al. (2012) [85], JADE	Jung-Poppe et al; Deawjaroen et al.; Falconer et al.	ADEs including ADRs and harm from errors	Japan	≥15 y/ medical, surgical wards, ICU	Prospective cohort	Development: n = 1,729; Validation: n = 1,730/ No	0.63 ± 0.03	NR (P)	NR (P)	8	NA
Sharif-Askari et al. (2014) [84], No name	Falconer et al. 2018	ADRs	UAE	Adults/ Renal ward	Prospective cohort	Development n = 512; Validation n = 1,000 (resamples)/ No	C-statistic 0.81 [0.76–0.87]	NR (SR)	Hosmer–Lemeshow P = 0.874	7	NA
Suggett (2017) [87], No name	Brady et al.	Pharmacist intervention	UK	Adult/All patients who had one or more pharmacist intervention (excluding CCU)	Retrospective cohort	n= 58,918 (75% modelling, 25% validation)/ Unclear	0.62	NR (P)	NR (SR)	19	NA
Tangiisuran et al. (2014) [62], BADRI Model	Brady et al; Deawjaron et al.; Jung-Poppe et al.; Stevenson et al.; Falconer et al.; Alshakrah et al.	ADRs	UK	Older population (≥65 y)/Geriatric and stroke wards	Prospective cohort	n= 690/ No	0.74 [0.68-0.79]	SN: 80% SP: 55%	Hosmer-Lemeshow test (p = 0.757)	5	Tangiisuran et al. (2014) [62]
Trivalle et al. (2011) [66], A geriatric score	Brady et al; Deawjaroen et al.; Stevenson et al; Falconer et al; Jung-Poppe et al.	ADEs	France	Older population (≥65 y)/Rehabili-tation centre, care unit, hospitals	Prospective cohort	n= 526/ Unclear	0.70 [0.65 -0.74]	NR (P)	NR (P)	3	NA
Urbina et al. (2014) [88], DRP risk Score	Brady et al; Jung-Poppe et al.; Deawjaroen; Falconer et al.	DRP	Spain	Adult/ Surgical and medical wards, internal medicine, surgical	Prospective cohort	Development: n = 7,202; Validation n = 3,598/ Yes	0.78 [0.76-0.79]	NR (P)	Hosmer Lemeshow’s test(P=0.131)	14	Ferrández et al. 2018 [99]
Winterstein et al. (2017) [89], C-score	Deawjaroen et al;	ADEs	US	Adult/ NR	Retrospective cohort	Development: n = 55,907; Validation: n = 100 (resampling)/NR (SR)	C-statistic = 0.84 [0.84-0.84]	NR (P)	NR (P)	310	NA
Zopf et al. (2008) [90], No name	Falconer et al.	ADRs	Germany	≥16 y/Internal medicine	Prospective cohort	n=907/ Yes	0.78 (model 1 – without labs) 0.80 (model 2 – with labs)	Model 1 SN:65%, SP: 80% Model 2: SN 64%, SP: 86%	NR (P)	5 (in the final model)	NA

*NR (SR)- Not reported by the systematic review; NR (P)- Not reported by the primary articles; SN (Sensitivity); SF (specificity).

ADR (Adverse Drug Reaction); ADE (Adverse Drug Event); ADDROP (the Adverse Drug Reaction Risk in Older Persons); AIME (the Adverse Inpatient Medication Event); ART (Assessment of Risk Tool); BADRI (Brighton ADR Risk); CCU (Cardiac Coronary Unit); C-score (Complexity Score); DART (Drug-Associated Risk Tool); DRP (drug related problems); ICU (Intensive Care Unit); JADE (the Japan Adverse Drug Events); ME (medication errors); MERIS (Medicine Risk Score); MOAT (Medicine Optimisation Assessment Tool); MRPs (Medication-Related Problems); PRIMSO (Predicting in-Hospital Significant Medication errors).

A prospective cohort study design was utilised by the more than half of the primary studies (n = 19, 59%) that developed and/or internally validated the risk prediction tool. Five primary studies had sufficient sample sizes or statistical power when considering the EPV [60,73,83,88,90], four were insufficiently powered [59,62,70,85], and the power of the 23 remaining studies was either unclear, not reported in the systematic reviews, or not applicable (e.g., descriptive study designs).

#### 3.3.1. Risk prediction tool performance.

The AUROC measured risk prediction tool discrimination in 12 studies [60,62,64,66,67,73,85–88,90,91] and C-statistic was used in six studies [59,75,82–84,89]. For the remaining tools, the systematic reviews did not specify or detail their discriminative ability. The discrimination of the models in the development and/or internal validation ranged from 0.65 to 0.84. Good discrimination was reported for the following 5 risk prediction tools: “Medicine Risk Score’ (MERIS), ‘Assessment of Risk Tool’ (ART), ’C-score’ and two unnamed tools developed by Zopf et al. and Sharif-Askari et al [84,90] (see Table 2). Another seven tools reached acceptable discrimination (i.e., The 80 + score’, ‘Adverse Inpatient Medication Event’ (AIME), ‘Predicting In-hospital Significant Medication Errors’ (PRISMORE), ‘Brighton Adverse Drug Reactions Risk’ (BADRI) tool, ‘GerontoNet ADR Risk Score’, ‘A geriatric score’, ‘DRP Risk Score’).

Sensitivity and specificity were reported for 13 tools with sensitivity scores ranging from 67% to 97% [77,79] and specificity from 30% to 88% [75,79]. The highest sensitivity score was reported for the ‘ADE & ADR Clinical Decision Rules’ tool, which achieved 97% sensitivity for identifying ADEs, with a corresponding specificity score of 40% for ADEs and 59% for ADRs [77]. The highest specificity score was reported for the ‘Drug Associated Risk Tool’ (DART version 2), which achieved 88% specificity for identifying patients without DRPs, with a corresponding sensitivity score of 67%.

Only eight (25%) of the 32 tools reported calibration data. Of these, six reported Hosmer–Lemeshow test results, all of which were statistically non-significant (p values ranging from 0.13 to 0.99), indicating acceptable calibration [62,65,82,84,88,91]. Two tools (‘MOAT’ and ‘PRISMOR’) had corrected calibration slopes, which suggested good calibration (0.88 and 0.93) respectively [75,83].

#### 3.3.2. External validation of the risk prediction tools.

Six of the 32 tools were externally validated as described within the systematic reviews (see table in S5 Table) including: ‘BADRI model’, ‘GerontoNet ADR Risk score’, DART (V2), ‘ADE and ADR Clinical Decision Rules’ and ‘DRP Risk Score’. No tool achieved good discriminative ability in the external validation, limiting clinical implementation, with the highest AUROC value of 0.75 for the tool ‘DRP Risk Score’. Sensitivity and specificity varied, with the ‘BADRI’ model achieving the highest sensitivity value of 84% (specificity 55%), and the ‘MERIS’ tool achieving the highest specificity score of 85% (sensitivity 57%).

### 3.4. Risk factors

Table 1 summarises the number of key risk factors reported in the systematic reviews, categorised into four groups (i.e., patient characteristics, clinical characteristics, medication classes, and other medication-related factors). Individual risk factors, including corresponding definitions used by the reviews (where available), are detailed in the table in S4 Table. Risk factors were often not clearly defined or operationalised within the studies. Furthermore, medication classes were inconsistently reported, with the Anatomical Therapeutic Chemical (ATC) Classification system inconsistently applied (e.g., 1^st^, 2^nd^, or 3rd ATC levels) and the point at which medication use was determined (e.g., on admission, during inpatient stay or at discharge) was often not clearly defined.

**Fig 3:**
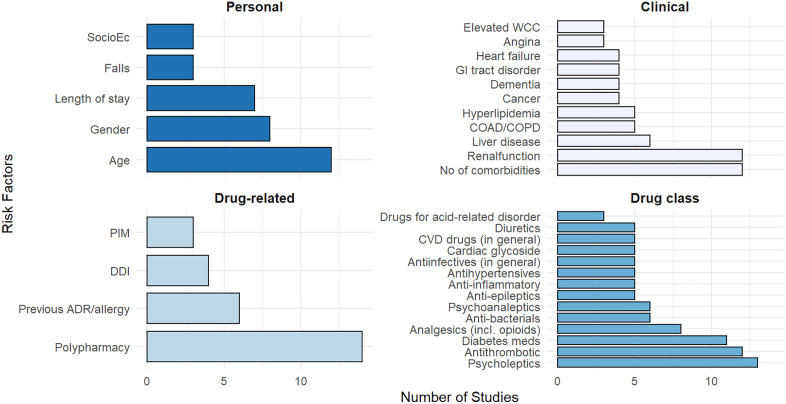
Common risk factors highlighted across the review studies. Fig 3 shows the common risk factors described by at least three reviews. SocioEc Socio Economic status; PIM Potentially Inappropriate Medications; DDI Drug-Drug Interactions; ADR Adverse Drug Reactions; WCC White Cell Count; GI Gastrointestinal; COAD/COPD Chronic Obstructive Airways Disease/Chronic Obstructive Pulmonary Disease; No Number; CVD Cardiovascular Disease.

Fig 3 shows the risk factors most commonly reported across the reviews. For the patient characteristics, age (i.e., increasing age or >60 years) was the most common identified factor (n = 12 reviews), followed by gender (n = 8) (most commonly female gender). The two most common clinical characteristics identified as risk factors were number of health conditions (e.g., ≥ 4) and impaired renal function (e.g., estimated glomerular filtration rate <30 mL/min or renal failure, both n = 12 reviews). Psycholeptics (e.g., antipsychotics, hypnotics/sedatives) were the most commonly implicated medication class (n = 13 reviews), followed by antithrombotic agents (n = 12 reviews). Other common medication-related factors were polypharmacy (n = 14 reviews), previous ADRs/allergies (n = 6 reviews), drug-drug interactions (n = 4 reviews) and potentially inappropriate medications (n = 3 reviews).

## 4. Discussion

This umbrella review synthesised information from 18 systematic reviews, encompassing 171 unique primary studies and 32 risk prediction tools, to assess their performance and identify risk factors for DRPs among hospitalised patients. However, none of the tools described in the systematic reviews appear to be automated or yet directly suitable for implementation in routine clinical practice. While five tools displayed good discriminatory performance in their internal validation, only one of them also showed good calibration. Furthermore, only six tools were externally validated. The risk factors commonly identified across these tools/models were often not clearly defined but highlight key areas relevant to consider when refining existing risk prediction tools.

### 4.1. Risk prediction tool performance

The identified tools often showed limited or incompletely reported performance in relation to discrimination, calibration, sensitivity, and specificity, all of which are important for clinical implementation. Only six of the 32 tools reported external validation, and only eight provided information on model calibration. In addition, only five tools reported sufficient statistical power based on events per variable (EPV), raising concerns about the validity of the reported AUROC values and, consequently, the predictive performance and clinical applicability of these tools. Of the 32 identified tools, two (i.e., BADRI [62], DRP Risk Score [88,99]) showed the highest potential for future clinical use based on their discriminatory ability, calibration and available external validation data. The BADRI tool focuses on older populations and adverse drug reactions (ADRs) as outcomes, whereas the DRP Risk Score is designed for general adult hospital inpatients to predict drug-related problems (DRPs). However, both appear to have an overall high risk of bias based on the PROBAST assessment [25] and could benefit from further external validation, clearer risk factor definitions and evaluation of their impact on patient prioritisation. Advancing evidence in these domains may support implementation in clinical practice, including improved usability and potential integration into digital or semi-automated workflows. These findings align with the conclusions of several systematic reviews included in this umbrella review [25,51,52].

Additionally, substantial heterogeneity existed in terms of predicted outcomes (e.g., ADRs, ADEs, medication errors, DRPs, readmission, or pharmacist intervention), patient populations, clinical settings, and number of predictors included across the tools. As a result, direct comparisons of model performance, including AUROC values, should be interpreted with caution. This heterogeneity also limits the generalisability of findings across hospital settings and patient populations. To our best knowledge, none of the identified tools are currently implemented in routine clinical practice. Nevertheless, this review compiled detailed information on existing tools, enabling stakeholders to identify tools for refinement, validation or future development, including potential AI-enabled approaches.

Advancements in AI systems and the increasing availability of large-scale electronic health records create new opportunities for applying AI in healthcare to enhance clinical practice, including risk prioritisation in pharmacy settings [100]. By combining existing risk prediction tools with deep learning techniques, it is possible to tailor these tools to specific hospital environments. Importantly, the findings of this review have a direct path to translation into clinical practice through the Automated Medicine Risk Review Program (AutoMedic) project, which is a federally funded Australian project that aims to develop and implement an AI-driven system to identify and prioritise inpatients for medication reviews as part of a clinical trial in tertiary hospitals.

### 4.2. Risk factors

Of the personal and clinical characteristics assessed, age, number of health conditions, and renal function were the most frequently reported risk factors relevant for patient prioritisation, as noted in 12 systematic reviews. However, it was often unclear whether risk factors (e.g., age, renal function) were treated as a continuous or categorical variables, and if so, which cut-points should be applied. This lack of standardisation challenges the usability in clinical practice and also limits the direct translation of reported risk factors into automated clinical decision support tools. While age is a well-recognised risk factor, frailty (which is particularly prevalent in older adults taking multiple medications), is often associated with drug-related problems and may therefore have greater clinical significance than chronological age [101]. However, frailty did not appear in the risk prediction tools identified in this review, possibly because there is currently no universally accepted measure of frailty [101] making it challenging to define and automate, or because individuals entering hospital may be experiencing sudden changes in their functional ability. In relation to comorbidities as a risk factor, many systematic reviews did not specify the number or type of health conditions most associated with DRPs. Future refinement of existing tools or development of new ones should aim to clearly define risk factors, including thresholds to improve clinical use and prediction.

Other commonly reported factors include gender (most commonly female), and length of hospital stay. These findings partially align with those of the umbrella review by Mihajlovic et al. [102], conducted in 2015, which identified older age and polypharmacy as the most frequent reported personal risk factor, followed by renal impairment, female sex, and cognitive decline across 11 reviews [102]. While longer length of stay was commonly associated with a higher risk of ADEs, it has limited utility for prioritising patients at admission, as it relies on retrospective data.

Common medication classes implicated in DRPs were consistent with other umbrella reviews which examined the incidence of, and patient characteristics associated with, ADE and preventable ADR in hospitalised adults, and identified anticoagulants, diabetes medications, and analgesics as most frequently associated with adverse outcomes [102,103]. Our umbrella review extends this knowledge by identifying psycholeptics (e.g., antipsychotics, hypnotics/sedatives) as additional classes associated with DRPs. This association likely reflects that many included studies focused on older adults, a population in which psycholeptics are commonly used to manage conditions such as behavioural and psychological symptoms of dementia, or delirium. Their inclusion among DRP-associated medication classes is expected given their adverse effect profile, and their more recent recognition as high-risk medications. For example, in certain Australian healthcare settings, psycholeptics, have been classified as a high-risk medications in recent years due to their potential to cause DRPs (e.g., prescribing errors) leading to significant harm [104–107]. In South Australia, this is reflected in the expansion of the high‑risk medication acronym APINCH to now include antipsychotics [104]. This is in line with international literature and professional organisations that have highlighted their potential to contribute to DRPs such as oversedation, worsening cognition, falls, and cerebrovascular events (particularly with antipsychotics), especially among older adults [108–112]. For example, the World Health Organization’s (WHO) Medication Without Harm global patient safety challenge prioritised high-risk medications as a key action area to reduce severe avoidable harm by 50% globally over five years [113]. Priority medications within this initiative include insulin, opioids, and anticoagulants, which overlap with the medications identified by our review. The WHO also recommends using software and tools that enable health professionals to identify and prioritise patients using high-risk medications as targets for interventions, supporting timely care and minimising DRPs [114]. However, a gap in evidence still exists as, it was unclear from the systematic reviews, whether the risk was related to the mere presence of a medication, or if it related to specific dosages/formulations or interactions with comorbidities. Other medication-related factors associated with DRPs included polypharmacy, history of previous ADRs or allergies, and drug-drug interactions. Together, these findings highlight the importance of automated risk prediction tools that can assist with timely identification of at-risk patients, deprescribe unnecessary medications, provide guidance on correct use, and manage potential adverse effects [115,116].

### 4.3. Strengths and limitations

Strengths of this umbrella review are that it summarises available risk prediction tools, reducing the need to navigate extensive literature, and confirms key risk factors while highlighting the need for clear definitions when developing or refining tools. We followed the PRISMA guidelines and conducted the review in accordance with the JBI Manual for Umbrella Reviews. All data screening and extraction was checked by a second reviewer, minimising extraction errors or misinterpretation. Nevertheless, an umbrella review is inherently reliant on the quality and methodological rigor of the included systematic reviews. Although a review-of-reviews approach was highly appropriate due to the large number of systematic reviews already published on this topic, there was considerable variability in their quality and reporting standards. Many of the included reviews either did not report or did not clearly describe their data extraction strategies (e.g., whether two reviewers were involved), which may have influenced the accuracy and consistency of data extraction in the original systematic reviews. Discrepancies in information regarding risk prediction tools were also observed across some reviews, necessitating cross-checking of primary studies to resolve conflicts. While we appraised the quality of the included systematic reviews, the quality assessment of primary studies was based on the evaluations conducted within those reviews. Another limitation relates to the often incomplete or inconsistent reporting of performance metrics for the risk prediction tools, as well as the lack of standardised definitions or detailed definitions for the included risk factors. In addition, the synthesis of risk factors was based on frequency of reporting across systematic reviews rather than quantitative pooling of effect sizes or formal assessment of the consistency and strength of associations across primary studies. Only systematic reviews that reported specific risk factors were included in this umbrella review. Consequently, the few identified reviews on AI-driven and electronic health record-based prediction models were excluded as the primary studies did not disclose risk factors, potentially due to proprietary reasons [26,27]. Therefore, a review specifically evaluating AI-based and machine learning prediction models could help identify relevant data-driven approaches that may complement and build upon the findings of the present review. Furthermore, we relied on the included systematic reviews to identify external validation studies, hence some additional external validations may not have been captured or have been published since the individual review search dates. Due to the qualitative nature of this umbrella review, a risk of bias assessment or sensitivity analysis was not possible.

## 5. Conclusion

This umbrella review provides a comprehensive and timely synthesis of existing risk prediction tools, including their performance and associated risk factors. Currently, no tool demonstrates strong discriminatory ability or has undergone extensive external validation across diverse populations to support routine clinical use in hospitals. Importantly, the risk factors identified in this review highlight key considerations that may assist in refining existing tool or developing new ones. Incorporating AI-based approaches could be considered to tailor implementation to the specific needs of hospital systems worldwide. Ultimately, an automated risk prediction tool suitable for routine clinical practice could not only optimize pharmacists’ time and interventions but also improve patient health outcomes.

## Supporting information

S1 Table Table of definitions. (PDF)

S2 Table Search strategy for academic literature (Ovid Medline). (PDF)

S3 Table Quality assessment of the systematic reviews (n = 20) based on the JBI Critical Appraisal checklist. (PDF)

S4 Table Individual factors associated with drug related problems within the four categories: Patient characteristics, clinical characteristics, medication classes, and other medication-related risk factors. (PDF)

S5 Table Summary of external validation studies of risk prediction tools. (PDF)

S1 Appendix Sheet 1: The full list of primary studies included in the systematic review. (XLSX)
