# Supplementary material for: Prediction tools to prioritise hospitalised adult patients at risk of drug related problems: An umbrella review
Source: PLOS Digit Health. 2026 Aug 6;5(8):e0001583. doi: 10.1371/journal.pdig.0001583 (PMC13446687; doi:10.1371/journal.pdig.0001583)
Supplement: Supplementary file 1 [file pdig.0001583.s001.pdf]

S1 Table: Table of definitions

Adverse drug event (ADE)	"Any untoward medical occurrence that may present during treatment with a pharmaceutical product but that does not necessarily have a causal relation to the treatment"[1, 2].
Adverse drug reactions (ADR)	"A response to a drug which is noxious and unintended and which occurs at doses normally used in man for prophylaxis, diagnosis, or therapy of disease, or the modification of physiological function"[1].<br><br>Adverse drug reactions can fall under the broader category of adverse drug event [3].
Drug related problem (DRP)	"Events involving drug therapy that actually or potentially interfaces with the patient experiencing an optimum outcome of medical care"[4].<br><br>The term DRP is an umbrella term that encompasses adverse drug events, adverse drug reactions and medication errors [2, 3, 5, 6].
Medication errors (ME)	"Any preventable event that may cause or lead to inappropriate medication use or patient harm while the medication is in the control of the health care professional, patient, or consumer"[7].

Reference:

1. World Health Organisation. Glossary of Patient Safety Concepts and References. 2009.
2. Falconer N, Barras M, Martin J, Cottrell N. Defining and classifying terminology for medication harm: a call for consensus. Eur J Clin Pharmacol. 2019;75(2):137-45.
3. Deawjaroen K, Sillabutra J, Poolsup N, Stewart D, Suksomboon N. Clinical usefulness of prediction tools to identify adult hospitalized patients at risk of drug-related problems: A systematic review of clinical prediction models and risk assessment tools. Br J Clin Pharmacol. 2022;88(4):1613-29.
4. Pharmaceutical Care Network Europe (PCNE). The PCNE Classification V 8.02. 2018. http://wwwpcneorg/upload/files/230_PCNE_classification_V8-02pdf. 2018.
5. van den Bemt PM, Egberts TC, de Jong-van den Berg LT, Brouwers JR. Drug-related problems in hospitalised patients. Drug Saf. 2000;22(4):321-33.
6. Al Hamid A, Ghaleb M, Aljadhey H, Aslanpour Z. A systematic review of hospitalization resulting from medicine-related problems in adult patients. Br J Clin Pharmacol. 2014;78(2):202-17.
7. National Coordinating Council for Medication Error Reporting and Prevention (NCC MERP). Taxonomy of Medication Errors 2019 [Available from: https://www.nccmerp.org/about-medication-errors.
