# Supplementary material for: Prediction tools to prioritise hospitalised adult patients at risk of drug related problems: An umbrella review
Source: PLOS Digit Health. 2026 Aug 6;5(8):e0001583. doi: 10.1371/journal.pdig.0001583 (PMC13446687; doi:10.1371/journal.pdig.0001583)
Supplement: Supplementary file 2 [file pdig.0001583.s002.pdf]

S2 Table: Search strategy for academic literature (Ovid Medline)

#	Query	Results from 19 Mar 2024
1	"Drug-Related Side Effects and Adverse Reactions"/	39,231
2	Medication Errors/	14,494
3	Near Miss, Healthcare/	371
4	("medication incident*" or "adverse drug event*" or "adverse drug reaction*" or "prescribing error*" or "dispensing error*" or "mediation administration error*" or "medication safety" or "drug related problem*" or "drug-related problem*").ti,ab,kf.	32,263
5	("adverse drug effect*" or "drug administration error*" or "Medicine* safety" or "medicine* related problem*" or "medication-related problem*" or "drug-related problem*" or "medication related problem*" or "drug related problem*" or "drug related harm*" or "medication related harm*" or "drug-related harm" or "medication-related harm" or "medicine* related harm" or "drug toxicity" or "poisoning by drug*").ti,ab,kf.	12,546
6	1 or 2 or 3 or 4 or 5	83,925
7	Medication Reconciliation/	1,590
8	"Medication Review"/	151
9	("medication review*" or "pharmac* service*" or "medication reconciliation" or "medicine* review" or "medicine* reconciliation").ti,ab,kf.	11,052
10	7 or 8 or 9	11,512
11	6 or 10	92,792
12	Triage/	15,335
13	Risk Assessment/	310,061
14	Risk/	128,742
15	Models, Statistical/	99,977
16	Artificial Intelligence/	44,509
17	Machine Learning/	37,765
18	Drug Therapy, Computer-Assisted/	1,693
19	(model* or predict* or tool* or risk or "artificial intelligence" or "computer reasoning" or "machine learning" or "patient priorit*" or triage or "decision support").ti,ab,kf.	8,145,162
20	12 or 13 or 14 or 15 or 16 or 17 or 18 or 19	8,325,885
21	exp Hospitals/	325,612
22	Academic Medical Centers/	20,954
23	Pharmacy Service, Hospital/	12,621
24	Inpatients/	30,832
25	Emergency Service, Hospital/	89,631
26	Surgery Department, Hospital/	4,565
27	Medication Systems, Hospital/	3,597
28	Patient Admission/	26,215
29	(inpatient* or hospital* or "clinical pharmac*").ti,ab,kf.	1,786,526

**30**	21 or 22 or 23 or 24 or 25 or 26 or 27 or 28 or 29	1,966,813
**31**	"Systematic Review"/	255,586
**32**	Meta-Analysis/	197,170
**33**	("systematic review" or metanalysis or "meta analysis" or meta-analysis).ti,ab,kf.	421,917
**34**	31 or 32 or 33	465,841
**35**	11 and 20 and 30 and 34	520
**36**	limit 35 to (english language and yr="2010-Current")	**470**
