# Supplementary material for: Prediction tools to prioritise hospitalised adult patients at risk of drug related problems: An umbrella review
Source: PLOS Digit Health. 2026 Aug 6;5(8):e0001583. doi: 10.1371/journal.pdig.0001583 (PMC13446687; doi:10.1371/journal.pdig.0001583)
Supplement: Supplementary file 3 [file pdig.0001583.s003.pdf]

S3 Table: Quality assessment of the systematic reviews (n=20) based on the JBI Critical Appraisal checklist

Author (year)	Q1	Q2	Q3	Q4	Q5	Q6	Q7	Q8	Q9*	Q10	Q11	Percentage
Alhawassi et al. (2014)	Y	Y	Y	Y	Y	U	U	N	N/A	Y	Y	70%
Alruqayb et al. (2021)	Y	Y	Y	Y	Y	Y	Y	Y	N/A	Y	Y	100%
Alshakrah et al. (2019)	Y	Y	Y	Y	Y	N	Y	Y	N/A	Y	Y	90%
AwangJihadi et al. (2023)	Y	Y	Y	Y	Y	Y	Y	Y	N/A	Y	Y	100%
Boeker et al. (2015)	Y	Y	Y	Y	Y	U	U	Y	Y	Y	Y	82%
Brady et al. (2020)	Y	Y	Y	Y	Y	N	N	N	N/A	Y	Y	70%
Danovitch et al. (2020)	Y	Y	Y	Y	Y	U	Y	Y	N/A	Y	Y	90%
Deawjaroen et al. (2022)	Y	Y	Y	Y	Y	Y	Y	Y	N/A	Y	Y	100%
Falconer et al. (2018)	Y	Y	Y	Y	Y	U	Y	Y	N/A	Y	Y	90%
Jennings et al. (2020)	Y	Y	Y	Y	Y	U	Y	Y	N/A	Y	Y	90%
Jung-Poppe et al. (2022)	Y	Y	Y	Y	N	U	U	Y	N/A	Y	Y	70%
Kharb et al. (2022) †	Y	Y	Y	Y	N	U	U	N	N/A	N	Y	50%
Lisha et al. (2017)	Y	Y	Y	Y	Y	U	U	N	N/A	Y	Y	70%
Mudigubba et al. (2018) †	Y	Y	Y	Y	U	N	U	N	N/A	N	U	40%
Patel et al. (2019)	Y	Y	Y	Y	Y	Y	Y	Y	N/A	Y	Y	100%
Sakiris et al. (2021)	Y	Y	Y	Y	Y	Y	U	Y	N/A	Y	Y	90%
Stevenson et al. (2014)	Y	Y	Y	Y	Y	Y	Y	Y	N/A	Y	Y	100%
Suggett et al. (2016)	Y	Y	Y	Y	N	U	U	Y	N/A	Y	Y	70%
Yadesa et al. (2021)	Y	Y	Y	Y	Y	Y	Y	Y	N/A	Y	Y	100%
Yiu et al. (2021)	Y	Y	Y	Y	Y	Y	U	Y	N/A	Y	Y	90%

Note. * Q9 was marked as not applicable (N/A) for all but one review, as the included systematic reviews primarily used narrative synthesis to identify risk factors for medication-related harm. † - Indicates that the study that excluded from the final evidence synthesis, for not meeting the minimum percentage of 70.

Yes – Criteria met, No- Criteria not met, U- Unclear, N/A – not applicable, the overall percentage is calculated by considering only the "yes" answers, which receive one point each, while all other answers receive zero points. A percentage (out of 100) is then calculated.

Q1: Is the review question clearly and explicitly stated?

Q2: Were the inclusion criteria appropriate for the review question?

Q3: Was the search strategy appropriate?

Q4: Were the sources and resources used to search for studies adequate?

Q5: Were the criteria for appraising studies appropriate?

Q6: Was the critical appraisal conducted by two or more reviewers independently?

Q7: Were there methods to minimize errors in data extraction?

Q8: Were the methods used to combine studies appropriate?

Q9: Was the likelihood of publication bias assessed?

Q10: Were the recommendations for policy and/or practice supported by the reported data?

Q11: Were the specific directives for new research appropriate?
