# Supplementary material for: Prediction tools to prioritise hospitalised adult patients at risk of drug related problems: An umbrella review
Source: PLOS Digit Health. 2026 Aug 6;5(8):e0001583. doi: 10.1371/journal.pdig.0001583 (PMC13446687; doi:10.1371/journal.pdig.0001583)
Supplement: Supplementary file 4 [file pdig.0001583.s004.pdf]

S4 Table: Individual factors associated with drug related problems within the four categories: Patient characteristics, clinical characteristics, medication classes, and other medication-related risk factors.

Author (year)	Patient characteristics	Clinical characteristics	Medication classes	Other medication-related risk factors
Alhawassi et al. (2014)	Increasing age, female, socioeconomic status, rural residential location, history of falls, limitation in ADLs	Comorbid burden, cardiovascular disease, diabetes mellitus, cancer, depression, dementia, liver disease, impaired renal function, hyperlipidaemia	Antithrombotic agents, non-steroidal anti-inflammatory and anti-rheumatic products, drugs used in diabetes, psycholeptics, antihypertensives	Drug-drug interaction, increased number of medications
Alruqayb et al. (2021)	Age & gender (not specified), length of stay	Degree of renal impairment	Antithrombotic agents, blood glucose lowering drug excluding Insulins, drugs used for peptic ulcer and gastro oesophageal reflux, vitamins, electrolytes with carbohydrates, antibacterials for systemic use, cardiac glycoside, lipid modifying agents	Drug-drug interaction, number of medications
Alshakrah et al. (2019)	Reason/type/time of admission, readmission, length of stay, age (not specified)	Hepatic impairment, comorbidity, renal impairment	Antithrombotic agents, non-steroidal anti-inflammatory and anti-rheumatic products, insulins and analogues, antipsychotics, antiepileptics, opioids, antidepressants, aminoglycoside antibacterials, anti-infectives (nil specified), direct acting antivirals, drugs used in cardiovascular disease, antineoplastic and immunomodulating agent, immunosuppressant, corticosteroids for systemic use, systemic drugs for obstructive airway disease	Drug requiring monitoring, total parenteral nutrition/nasogastric tube, high-cost medicine, number of unlicensed medicine & intravenous medicine, high-risk medicine, polypharmacy
Awang Jihadi et al. (2023)	Age (> 50 years), female	Comorbidity	Antithrombotic agents, blood glucose lowering drug excluding Insulin, antibacterials for systemic use, antihypertensive	Number of medications/polypharmacy
Boeker et al. (2015)	Age (not specified)	Nil	Antithrombotic agents, hypnotics/sedatives, antibacterials for systemic use, diuretics, antihypertensives	Polypharmacy

Brady et al. (2020)	Age (not specified), female, social support, history of falls, length of stay, recent hospitalisation	Comorbidity, heart failure, angina, gastrointestinal disorder, chronic obstructive airway disease, malignant disease, liver disease, pulmonary disease, reduced renal function, elevated white cell count, abnormal potassium levels, INR levels	Antithrombotic agents, drugs used in diabetes, drugs used for peptic ulcer and gastro oesophageal reflux, antipsychotics, antidepressants, anti-infectives (nil specified), cardiac glycoside, immunosuppressants, general high-risk medications (e.g. STOPP (Screening Tool of Older Persons' Prescriptions) medication)	Previous adverse drug reactions or allergies, number of medications/polypharmacy, potentially inappropriate medicines, recent changes in medicines
Danovitch et al. (2020)	Age (<60 years), female, higher income,	Higher Charlson comorbidity index, obesity, chronic obstructive airway disease, substance use disorder, asthma, renal disease	Hypnotics/sedatives, drugs affecting the nervous system (central nervous depressants)	Patients exposed to both opioids and sedatives
Deawjaroen et al. (2022)	Age (not specified),	Comorbidity, renal impairment	NA	Previous adverse drug reactions or allergies, number of medications, high-risk medicine
Falconer et al. (2018)	Age (>60 years), treatment initiated before admission, Length of stay (>/= 12 days)	Burden of illness more than 4 comorbid conditions or number of diagnoses, heart failure, angina, gastrointestinal disorder, chronic obstructive airway disease, cancer, liver disease, dementia, musculoskeletal/connective tissues, kidney and urinary tract infections, nervous system, vascular disease, hemiplegia, consciousness, dyspnoea, raised body	Antithrombotic agents, drugs used in diabetes, drugs used in constipation, antipsychotics, anti-infectives (nil specified), drugs used in cardiovascular disease, cardiac glycoside, systemic hormonal preparations excluding Sex hormones and insulins, medications for sensory organs	Previous adverse drug reactions or allergies, number of medicines (Varied between >8-10), number of STOPP (Screening Tool of Older Persons' Prescriptions) medicines, potentially inappropriate medicines

		temperature, renal disease, hyperlipidaemia, elevated white cell count, potassium levels, thrombocyte count, high c-reactive protein, serum albumin less than 3.5g/dL, low erythrocyte count		
Jennings et al. (2020)	Nil	Comorbidity (mean/median of ≥ three chronic conditions)	Antithrombotic agents, antianemic preparations, anti-inflammatory/antirheumatic products, drugs used in diabetes, drugs used for peptic ulcer and gastro oesophageal reflux, drugs used in constipation, mineral supplements, antipsychotics, hypnotics/sedatives, antiepileptics, analgesics, antidepressants, anti-dementia drugs, antibacterials for systemic use, cardiac glycoside, agents acting on renin-angiotensin system, peripheral vasodilators, calcium channel blockers, diuretics, antihypertensives, adrenergic (inhalants)	mean/median of ≥ five daily medications
Jung-Poppe et al. (2022)	Age (> 60 years)	Comorbidity, renal function less than 30 mL/minute	Psycholeptics, antiepileptics, analgesics	Number of drugs ≥8
Lisha et al. (2017)	Transport of a patient for diagnostic or surgical intervention outside of intensive care unit	Renal dysfunction or kidney injury, thrombocytopenia	Antithrombotic agents, drugs used in diabetes, hypnotics/sedatives, analgesics, anti-infectives (nil specified), drugs used in cardiovascular disease, diuretics	Polypharmacy – with more than 10 drugs, administered intravenous medication, off-label medication use
Patel and Patel (2019)	Nil	Nil	Antithrombotic agents, non-steroidal anti-inflammatory and anti-rheumatic products, antineoplastic agents	Drug-drug interaction (such as combinations of NSAIDs, antithrombotics, and corticosteroids, combinations of cephalosporins with fluroquinolones and/or

				penicillins, combination of loop diuretics with renin-angiotensin system inhibitors, and gentamicin),
Sakiris et al. (2021)	Nil	Nil	Antipsychotics, other (psychotropics), drugs used in diabetes, antiepileptics, analgesics, anti-depressants, antibacterials for systemic use, drugs used in cardiovascular disease, diuretics, antihypertensives	Patients taking more than 5 drugs
Stevenson et al. (2014)	Length of stay (more than 12 days)	Angina, gastrointestinal disorder, chronic obstructive airway disease, renal failure, hyperlipidaemia, elevated white cell count	Antithrombotic agents, non-steroidal anti-inflammatory and anti-rheumatic products, psycholeptic, analgesics, antidepressants, cardiac glycoside, angiotensin converting enzyme inhibitors (plain)	Previous ADR, polypharmacy
Suggett and Marriott, (2016)	Age (> 65 years), female, length of stay (nil specified)	Comorbidity, liver disease, poor renal function (nil specified)	Antithrombotic agents, insulins and analogues, drugs affecting the nervous system (central nervous system agents), antiepileptics, opioids, anti-infectives (nil specified), drugs used in cardiovascular disease, diuretics, antineoplastic and immunomodulating agents, corticosteroids for systemic use	History of drug allergy or sensitivity, patient compliance issues
Yadesa et al. (2021)	Female, rural residence, previous falls, emergency admission, smoking	Four or more comorbid conditions (or higher Charlson comorbidity index), heart failure, atrial fibrillation, diabetes mellitus, cancer, dementia, liver disease, renal disease, hyperlipidaemia	Drugs used in diabetes, vitamins	Previous ADR, concurrent use of five or more medications, potentially inappropriate medications
Yiu et al. (2021)	Age (>65 years), male	higher Charlson Comorbidity Index (CCI) or Elixhauser Comorbidity Score, congestive heart failure, shock, acute myocardial infarction, gastrointestinal haemorrhage,	Hypnotics/sedatives, opioids	Drug-drug interaction (such as coadministration of sedating medicines)

		chronic obstructive airway disease, depression, dementia, anxiety, mood disorders, delirium		
