# Supplementary material for: Prediction tools to prioritise hospitalised adult patients at risk of drug related problems: An umbrella review
Source: PLOS Digit Health. 2026 Aug 6;5(8):e0001583. doi: 10.1371/journal.pdig.0001583 (PMC13446687; doi:10.1371/journal.pdig.0001583)
Supplement: Supplementary file 5 [file pdig.0001583.s005.pdf]

S5 Table: Summary of external validation studies of risk prediction tools

Author (year), Tool name	Systematic Reviews	External Validation				
		Author (year)	Sample Size	Discrimination AUROC (95% CI)	Sensitivity	Specificity
Tangiisuran et al. (2014)[1], **BADRI Model**	Brady et al.; Deawjaroen et al; Jung-Poppe; Stevenson et al.; Falconer et al.	Tangiisuran et al. (2014)[1]	n= 483	0.73 (0.66-0.80)	84%	55%
Onder et al. (2010)[2], **GerontoNet ADR Risk Score**	Brady et al; Deawjaroen et al; Jung-Poppe et al; Stevenson et al; Falconer et al.	A: Onder et al (2010)[2]<br>B: O'Conner et al (2012)[3];<br>C: Petrovic et al (2017)[4];	A: n= 483<br>B: n= 513<br>C: n=1075	A: 0.70 (0.63–0.78)<br>B: 0.62 (0.57–0.68)<br>C: 0.64 (0.55–0.74)	A: NR (P)<br>B: NR (SR)<br>C: NR (SR)	A: NR (P)<br>B: NR (SR)<br>C: NR (SR)
Saedder et al. (2016)[5], **MERIS**	Brady et al.; Deawjaroen et al; Jung-Poppe; Alshakrah et al	A: Bonnerup et al. (2016)[6]<br>B: Høj et al., (2021)[7]	A: n= 103<br>B: NR (SR)	A: NR (SR)<br>B: NR (SR)	A: 57%<br>B: NR (SR)	A: 85%<br>B: NR (SR)
Kaufmann et al. (2018)[8], **DART V2**	Brady et al.; Deawjaroen et al.	Staempfli et al. 2018[9]	n= 110	NR (P)	NR (P)	NR (P)
Hohl et al. (2012)[10], **ADE & ADR Clinical Decision rules**	Brady et al; Jung-Poppe et al	A: Hohl et al. (2017)[11] (impact study)<br>B: Hohl et al. (2018)[12] (external validation)	A:NR (SR)<br>B: NR (SR)	A:NR (P)<br>B: NR (SR)	A:NR (P)<br>B: NR (SR)	A: NR (P)<br>B: NR (SR)
Urbina et al. (2014)[13], **DRP Risk Score**	Brady et al; Jung-Poppe et al.; Deawjaroen; Falconer et al.	Ferrández et al. 2018[14]	n= 3598	0.75 (0.75-0.76)	77.7%	60.9%

Note: NR (SR)- Not reported by the systematic review; NR (P)- Not reported by the primary articles

References

1. Tangiisuran B, Scutt G, Stevenson J, et al. Development and validation of a risk model for predicting adverse drug reactions in older people during hospital stay: Brighton Adverse Drug Reactions Risk (BADRI) model. *PLoS One*. 2014;9(10):e111254. doi:10.1371/journal.pone.0111254
2. Onder G, Petrovic M, Tangiisuran B, et al. Development and Validation of a Score to Assess Risk of Adverse Drug Reactions Among In-Hospital Patients 65 Years or Older: The GerontoNet ADR Risk Score. *Archives of Internal Medicine*. 2010;170(13):1142-1148. doi:10.1001/archinternmed.2010.153
3. O'Connor MN, Gallagher P, Byrne S, O'Mahony D. Adverse drug reactions in older patients during hospitalisation: are they predictable? *Age Ageing*. Nov 2012;41(6):771-6. doi:10.1093/ageing/afs046
4. Petrovic M, Tangiisuran B, Rajkumar C, van der Cammen T, Onder G. Predicting the Risk of Adverse Drug Reactions in Older Inpatients: External Validation of the GerontoNet ADR Risk Score Using the CRIME Cohort. *Drugs Aging*. Feb 2017;34(2):135-142. doi:10.1007/s40266-016-0428-4
5. Saedder EA, Lisby M, Nielsen LP, et al. Detection of Patients at High Risk of Medication Errors: Development and Validation of an Algorithm. *Basic Clin Pharmacol Toxicol*. Feb 2016;118(2):143-9. doi:10.1111/bcpt.12473
6. Bonnerup DK, Lisby M, Sædder EA, et al. Risk of prescribing errors in acutely admitted patients: a pilot study. *Int J Clin Pharm*. Oct 2016;38(5):1157-63. doi:10.1007/s11096-016-0345-y
7. Høj K, Pedersen HS, Lundberg ASB, Bro F, Nielsen LP, Sædder EA. External validation of the Medication Risk Score in polypharmacy patients in general practice: A tool for prioritizing patients at greatest risk of potential drug-related problems. *Basic & Clinical Pharmacology & Toxicology*. 2021/10/01 2021;129(4):319-331. doi:https://doi.org/10.1111/bcpt.13636
8. Kaufmann CP, Stämpfli D, Mory N, Hersberger KE, Lampert ML. Drug-Associated Risk Tool: development and validation of a self-assessment questionnaire to screen for hospitalised patients at risk for drug-related problems. *BMJ Open*. Mar 9 2018;8(3):e016610. doi:10.1136/bmjopen-2017-016610
9. Stämpfli D, Boeni F, Gerber A, et al. Assessing the ability of the Drug-Associated Risk Tool (DART) questionnaire to stratify hospitalised older patients according to their risk of drug-related problems: a cross-sectional validation study. *BMJ Open*. Jun 27 2018;8(6):e021284. doi:10.1136/bmjopen-2017-021284
10. Hohl CM, Yu E, Hunte GS, et al. Clinical decision rules to improve the detection of adverse drug events in emergency department patients. *Acad Emerg Med*. Jun 2012;19(6):640-9. doi:10.1111/j.1553-2712.2012.01379.x
11. Hohl CM, Partovi N, Ghement I, et al. Impact of early in-hospital medication review by clinical pharmacists on health services utilization. *PLoS One*. 2017;12(2):e0170495. doi:10.1371/journal.pone.0170495
12. Hohl CM, Badke K, Zhao A, et al. Prospective Validation of Clinical Criteria to Identify Emergency Department Patients at High Risk for Adverse Drug Events. *Acad Emerg Med*. Sep 2018;25(9):1015-1026. doi:10.1111/acem.13407

13. Urbina O, Ferrández O, Grau S, et al. Design of a score to identify hospitalized patients at risk of drug-related problems. *Pharmacoepidemiol Drug Saf*. Sep 2014;23(9):923-32. doi:10.1002/pds.3634
14. Ferrández O, Grau S, Urbina O, Mojal S, Riu M, Salas E. Validation of a score to identify inpatients at risk of a drug-related problem during a 4-year period. *Saudi Pharm J*. Jul 2018;26(5):703-708. doi:10.1016/j.jsps.2018.02.020
