# Supplementary material for: Prediction tools to prioritise hospitalised adult patients at risk of drug related problems: An umbrella review
Source: PLOS Digit Health. 2026 Aug 6;5(8):e0001583. doi: 10.1371/journal.pdig.0001583 (PMC13446687; doi:10.1371/journal.pdig.0001583)
Supplement: Supplementary file 6 [file pdig.0001583.s008.docx]

**Author response letter for PLOS Digital Health submission**

Manuscript title: Prediction tools to prioritise hospitalised adult patients at risk of drug related problems: An umbrella review.

Manuscript ID: PDIG-D-26-00198

We thank the Editor and all Reviewers for their valuable time and constructive comments. All Reviewers comments and questions are addressed below. Specific additions to the text are italicised and highlighted in yellow in the responses below. Further, all changes made to the manuscript are highlighted in yellow in the manuscript file.

**Editor Comments:**

**Journal Requirements:**

1. Please upload separate figure files in .tif or .eps format. Also, remove the figures from your manuscript file but keep the legends.

For more information about figure files please see our guidelines:

[https://journals.plos.org/digitalhealth/s/figures](https://url.au.m.mimecastprotect.com/s/p4mzCoV140cr0yN19uVi3TpbcuI?domain=journals.plos.org) 
[https://journals.plos.org/digitalhealth/s/figures#loc-file-requirements](https://url.au.m.mimecastprotect.com/s/vyoHCp815qInBRrJkfGsBTG15kW?domain=journals.plos.org)

Response: Apologies for this oversight, we have now removed the figures from the manuscript and have kept legends directly after the paragraph in which they were first cited.

2. We have noticed that you have uploaded Supporting Information files, but you have not included a list of legends. Please add a full list of legends for your Supporting Information files after the references list.

Response: We thank you for this comment. We now have added a full list of legends for the supporting information files after the reference list.

If the reviewer comments include a recommendation to cite specific previously published works, please review and evaluate these publications to determine whether they are relevant and should be cited. There is no requirement to cite these works unless the editor has indicated otherwise.

**Reviewers' Comments**

**Reviewer #1:**

1. There is a lot of variation in how reviews define "acceptable" and "good" discrimination. This inconsistency can affect how the 32 tools are assessed.

Response: We agree that tool performance, including discrimination, is an important measure. We have outlined in the Methods section, based on past literature the cut-off values used for the AUROC to assess discrimination (p.11): “*We considered an AUROC of 0.70-0.80 to represent acceptable discrimination and >0.80 indicated good discrimination (25*).”). In addition, we now acknowledge that other performance metrics, such as calibration, are also important when evaluating prediction tools. We have further included a statement regarding events per variable (EPV) in relation to AUROC estimates and emphasised that caution is required when comparing performance metrics across tools due to differences in model characteristics and development methods.

Discussion (p. 32): “*In addition, only five tools reported sufficient statistical power based on events per variable (EPV), raising concerns about the validity of the reported AUROC values and, consequently, the predictive performance and clinical applicability of these tools*.”

Discussion (p. 33): “*Additionally, substantial heterogeneity existed in terms of predicted outcomes (e.g., ADRs, ADEs, medication errors, DRPs, readmission, or pharmacist intervention), patient populations, clinical settings, and number of predictors included across the tools. As a result, direct comparisons of model performance, including AUROC values, should be interpreted with caution.”*

2. Only two tools showed good potential for clinical use, but the manuscript does not explain clearly which populations the validation worked for and which it did not.

Response: Thank you for highlighting this point. We have now added a sentence to the Discussion section outlining the patient populations and outcome measures used in the two tools with the greatest potential for clinical use, namely BADRI and DRP Risk Score. This clarification provides additional context regarding the populations in which these tools were developed and validated, as well as the outcomes they were designed to predict.

Discussion (p. 32,33): “*The BADRI tool focuses on older populations and adverse drug reactions (ADRs) as outcomes, whereas the DRP Risk Score is designed for general adult hospital inpatients to predict drug-related problems (DRPs).”*

3. Although "reduced renal function" is an important factor, the reviews often do not provide a clear definition, such as specific eGFR thresholds. This lack of clarity makes it harder to create automated alerts.

Response: We agree that the lack of clear definitions for certain risk factors, such as reduced renal function, limits their usability and direct applicability for automated implementation. We have therefore expanded upon this point in Section 4.2 (p.34) of the Discussion: “*However, it was often unclear whether risk factors (e.g. age, renal function) were treated as a continuous or categorical variables, and if so, which cut-points should be applied. This lack of standardisation limits the direct translation of reported risk factors into automated clinic decision support tools…. Future refinement of existing tools or development of new ones should aim to clearly define risk factors, including thresholds to improve clinical use and prediction.”*

We have also acknowledged this in the discussion under limitations (p 37): “*Another limitation relates to the often incomplete or inconsistent reporting of performance metrics for the risk prediction tools, as well as the lack of standardised definitions or detailed definitions for the included risk factors”.*

4. The overall clinical classification accuracy (CCA) is low at 3.47%, but there is significant overlap between the findings of different reviews. This suggests that some results may be over-represented.

Response: We agree that, despite the low CCA, several systematic reviews reported similar conclusions regarding the clinical utility of the tools as well as similar risk factors. We have extracted and synthesised risk prediction tools at the level of the individual tool to minimise overrepresentation of specific tools. However, we acknowledge that the risk factor synthesis is narrative in nature, and that the reported frequencies should be interpreted cautiously, as they reflect the frequency of reporting across systematic reviews rather than independent quantitative confirmation of the strength or consistency of associations. We have accordingly added clarification of this in both the Methods and Discussion sections.

Method p (12): “*Risk factor counts were based solely on the number of systematic reviews highlighting a given factor and should therefore be interpreted as a descriptive measure of reporting frequency rather than the relative importance, weighting, or effect size of the factor within individual prediction models. Furthermore, as overlap in primary studies was present across the included reviews, the reported frequencies may not represent fully independent lines of evidence*.”

Discussion p. (37): “*In addition, the synthesis of risk factors was based on frequency of reporting across systematic reviews rather than quantitative pooling of effect sizes or formal assessment of the consistency and strength of associations across primary studies.”*

5. In the Introduction, after discussing automated prioritization tools. It is suggested to refer from the paper: “Analyzing cybersecurity risks and threats in IT infrastructure based on the NIST framework “. This reference is important for discussing the security and risk management needed when implementing "automated, AI-enabled tools" in hospitals. It supports the manuscript by providing a basis for identifying weaknesses in the digital infrastructure that will support these drug-related problem (DRP) prediction tools.

Response: We agree that security and risk management are important aspects to consider when implementing automated or AI-enabled systems in hospital settings. However, based on comments from Reviewers 2 and 3, we reduced the emphasis on AI and machine learning within the current manuscript, as none of the tools included in this review directly utilised AI-based approaches. Consequently, we have chosen to limit the discussion of AI-enabled tools to future directions. Nevertheless, we appreciate this suggestion and will consider incorporating a more detailed discussion on cybersecurity and digital infrastructure in forthcoming manuscripts linked to the broader AutoMedic project.

6. The authors need to clarify the area under the receiver operating characteristic curve (AUROC) thresholds used. While a range of 0.70 to 0.80 is described as "acceptable," the authors should explain why tools below this threshold are still included in clinical summaries.

Response: We also acknowledge that other performance metrics (e.g., sensitivity, specificity, and calibration) as well as practical considerations such as ease of use play an important role in determining the clinical utility of a tool. The categorisation of AUROC values between 0.70 and 0.80 as “acceptable” was based on commonly applied conventions in the literature [1], rather than as a strict criterion for inclusion or exclusion. AUROC values were also not used to determine eligibility for inclusion of tools in this review. We have now accordingly modified the method section to make this clearer. In addition, we have added calibration values where available in Table 2 and have expanded the discussion in relation of events per variable (EPV), as this can influence the robustness and interpretation of reported AUROC values (see response to Comment 1).

Method section 2.5 Data Extraction page 10: *“For a tool to be suitable for clinical implementation, adequate performance in both internal and external validation is required. In line with recommendations for reporting and appraising models (i.e., PROBAST [35]), we extracted measures of discrimination and calibration to assess model performance. Discrimination was assessed using the area under the receiver operating characteristic curve (AUROC) or concordance (C) statistic, which reflects the ability of a model to distinguishes between patients who will and will not experience the outcome of interest (e.g. ADE). We considered an AUROC of 0.70-0.80 to represent acceptable discrimination and >0.80 indicated good discrimination [25]. Sensitivity (i.e., the tool’s ability to correctly identify patients who will experience the outcome) and specificity (i.e., ability to correctly identify those who will not experience the outcome) were also extracted. Another key measure of predictive performance is a model’s calibrations, which refers to the agreement between the predicted probability by a model and the actual outcomes. Perfect calibration is indicated by an intercept approaching 0 and a slope approaching 1, whereas a statistically significant Hosmer–Lemeshow test suggests poor model calibration [37].”*

7. A clearer breakdown is needed to explain why current tools are not suitable for regular clinical use. Are the problems due to data silos, a lack of real-time integration with the electronic medical record (EMR), or low sensitivity?

Response: Thank you for raising this point. We have made adjustments throughout the manuscript to further support our statement that none of the identified tools appear to be in routine clinical use or are yet directly suitable for implementation in practice. Tool performance (i.e. discrimination, calibration, sensitivity, and specificity), as well as internal and external validation, are key factors determining clinical applicability. In addition, usability and implementation considerations also play an important role. The changes made in the Methods section related to this issue are outlined in response to Comment 6 above.

The Discussion (p. 32) now reads: “*The identified tools often showed limited or incompletely reported performance in relation to discrimination, calibration, sensitivity, and specificity, all of which are important for clinical implementation. Only six of the 32 tools reported external validation, and only eight provided information on model calibration. In addition, only five tools reported sufficient statistical power based on events per variable (EPV), raising concerns about the validity of the reported AUROC values and, consequently, the predictive performance and clinical applicability of these tools. Of the 32 identified tools, two (i.e. BADRI [62], DRP Risk Score [88,99] showed the highest potential for future clinical use based on their discriminatory ability, calibration and available external validation data…. .However, both appear to have an overall high risk of bias based on the PROBAST assessment [25] and could benefit from further external validation in different patient populations, clearer risk factor definitions and evaluation assessment of their impact on patient prioritisation.”*

8. The "Author Summary" and "Introduction" should be expanded to better connect existing risk factors with the creation of AI-enabled automated tools.

Response: We acknowledge that the relationship between identified risk factors and risk prediction tools and potential AI-enabled automated tools should be interpreted with caution, as the present review did not specifically target AI-based prediction models and excluded some reviews in this area because they did not report individual risk factors (see also our response to Reviewer 3, Comment 3). In line with feedback from Reviewer 2 and Reviewer 3, we have revised the manuscript to ensure that AI-related statements are framed more carefully and do not extend beyond the scope of the included evidence but rather focus on suggestions for future research. To maintain consistency with the scope of this review, we have therefore chosen to not further expand on AI-enabled tools in the other sections.

9. Consistency in terminology is important. For example, "ADR," "ADE," and "DRP" are used interchangeably. A table of definitions in the Introduction would enhance understanding.

Response: Thank you for raising this point. In response, we have now added a line in the introduction (p.7), which reads “Definitions of these terms can be found within the table in S1 Table”.

A table containing the list of terms such as “ADR”, “ADE” and “DRP” and its definitions have been added to the supporting files.

**Reviewer #2:** The manuscript addresses an important and clinically relevant topic, and the authors have applied an appropriate methodological framework, including adherence to PRISMA guidelines, PROSPERO registration, and the use of Joanna Briggs Institute appraisal tools. The literature search appears comprehensive, and the inclusion of a large number of systematic reviews and primary studies provides a valuable overview of the current evidence base. However, several issues limit the strength and clarity of the manuscript.

1. First, the scope of the study is overly broad. The manuscript attempts to simultaneously identify prediction tools, evaluate their performance, and summarise risk factors. As a result, none of these components are explored in sufficient depth. A clearer prioritisation of objectives would improve the overall coherence and analytical strength of the paper.

Response: We acknowledge that this umbrella review addresses several aspects, including prediction tools, therein performance, and associated risk factors.

The primary focus of the review was always the identification of available prediction tools and the evaluation of their predictive performance. The synthesis of risk factors was intended as a secondary component to contextualise the tools, rather than a detailed quantitative re-analysis of individual risk factors from primary studies. As recent systematic reviews have already evaluated specific risk factors, we considered a narrative overview more appropriate within the scope of this umbrella review. To improve clarity, we have now explicitly stated that the risk factor synthesis was narrative in nature throughout the manuscript have revised the aim as follows (p.8):

“*The primary aim of this umbrella review is to identify available risk prediction tools and summarise their predictive performance, while the secondary aim is to narratively summarise key risk factors, including patient characteristics and medication-related factors, that are important for minimising DRPs in hospitalised patients.”*

2. Second, the conclusions are somewhat stronger than what the data can support. In particular, the statement that no tools are suitable for routine clinical practice is not adequately justified, as the manuscript does not define explicit criteria for clinical suitability nor systematically assess clinical utility. A more cautious and clearly defined interpretation would be appropriate.

Response: Thank you for this comment, and we agree that the original wording required further refinement. We have revised the manuscript to make this statement more cautious, and emphasising that, to our knowledge, none of the identified tools are currently in broad routine clinical use. We have updated both the Methods and Discussion sections to better clarify that clinical applicability is determined by multiple performance measures (including discrimination, calibration, sensitivity, and specificity) as well as evidence from internal and external validation. We have further highlighted the importance of clinical utility, including the definition and operationalisation of risk factors.

Please also see our response to Reviewer 1, Comment 7, which outlines the related changes made in the Discussion section.

3. Third, the evaluation of prediction tools is limited primarily to discrimination metrics such as AUROC, without consideration of other important aspects of model performance, including calibration and clinical usefulness. This restricts the ability to draw meaningful conclusions about real-world applicability.

Response: We agree and have now also extracted information about the calibration of the individual tools and have also discussed the risk of bias assessment in the development. As outlined in the comment above we have now accordingly adjusted the method and results section as well as the discussion.

Method section 2.4 p.10*:” In addition, we extracted whether the systematic reviews applied a specific appraisal tool for prediction models, such as PROBAST (Prediction model Risk Of Bias ASsessment Tool), to assess risk of bias and applicability of the prediction models [35].”*

*Method section 2.5 p10-11: “For a tool to be suitable for clinical implementation, adequate performance in both internal and external validation is required. In line with recommendations for reporting and appraising models (i.e. PROBAST), we extracted measures of discrimination and calibration to assess model performance. Discrimination was assessed using the area under the receiver operating characteristic curve (AUROC) or concordance (C) statistic, which reflects the ability of a model to distinguishes between patients who will and will not experience the outcome of interest (e.g. ADE). We considered an AUROC of 0.70-0.80 to represent acceptable discrimination and >0.80 indicated good discrimination [25]. Sensitivity (i.e., the tool’s ability to correctly identify patients who will experience the outcome) and specificity (i.e., ability to correctly identify those who will not experience the outcome) were also extracted. Another key measure of predictive performance is a model’s calibration, which refers to the agreement between the predicted probability by a model and the actual outcomes. Perfect calibration is indicated by an intercept approaching 0 and a slope approaching 1, whereas a statistically significant Hosmer–Lemeshow test suggests poor model calibration [37].”*

4. Fourth, the synthesis of risk factors is largely descriptive, based on frequency of reporting across reviews, without consideration of effect size, consistency, or methodological quality. This approach should be interpreted with caution and more clearly framed as a narrative summary rather than a quantitative synthesis.

Response: We agree that the narrative nature of risk factor synthesis is needed to be more clearly articulated. We have now consistently referred to the risk factor component throughout the manuscript as a narrative summary rather than a quantitative synthesis. Furthermore, we have added clarification in Section 2.6, p.12 (Data Synthesis and study overlap) of the Methods to emphasize that the reported frequencies should be interpreted with caution:

“*Risk factor counts were based solely on the number of systematic reviews highlighting a given factor and should therefore be interpreted as a descriptive measure of reporting frequency rather than the relative importance, weighting, or effect size of the factor within individual prediction models. Furthermore, as overlap in primary studies was present across the included reviews, the reported frequencies may not represent fully independent lines of evidence.”*

5. Finally, the discussion of artificial intelligence is speculative and not directly supported by the data presented in the review. This section should be toned down or more clearly presented as a future perspective.

Response: Thank you for raising this, we acknowledge that some AI tools do already exist but were not included in the current review, as individual risk factors were not outlined and that some of the sections needed to better reflect this as a future perspective. We have accordingly adjusted the manuscript as outline in detail in our response to Reviewer 3, comment 3.

In summary, while the manuscript has clear strengths and addresses an important topic, substantial revision is required to improve focus, methodological depth, and alignment between results and conclusions.

Response: Thank you very much for this constructive summary. We have addressed all raised points throughout the manuscript, with the aim of improving its focus, methodological clarity, and the alignment between results and conclusions.

**Reviewer #3:** This umbrella review synthesizes 18 systematic reviews encompassing 171 unique primary studies and 32 risk prediction tools for drug-related problems (DRPs) in hospitalized adults. The topic is timely and practically important: pharmacists need evidence-based tools for patient prioritization, and this review provides a useful landscape analysis. The umbrella review design is well-suited to the mature state of this literature, and the authors followed JBI methodology with a registered protocol on PROSPERO. The overlap analysis, quality appraisal, and detailed tool-level extraction in Table 2 are strengths.
However, some issues require attention before the manuscript is suitable for publication. I have organized these into major issues (which require revision) and minor issues (which could be addressed through additional limitation language or brief additions). I note that my observations are about strengthening an already useful contribution, not about fundamental flaws in the study design.

Major Issues

1. Absence of prediction model-specific risk-of-bias assessment (PROBAST).
The authors used the JBI critical appraisal checklist for systematic reviews to assess the quality of the included reviews. This is appropriate for appraising the reviews as reviews. However, the primary subject matter of this umbrella review is the performance of prediction models, and the manuscript does not discuss whether the included systematic reviews themselves used any prediction model-specific risk-of-bias tool to appraise their primary studies. PROBAST (Prediction model Risk Of Bias ASsessment Tool) is the consensus standard for assessing risk of bias and applicability in prediction model studies. This is directly relevant to interpreting the AUROC values in Table 2. The authors should: (a) report whether any of the six systematic reviews that evaluated risk prediction tools used PROBAST or an equivalent prediction model-specific tool; and (b) discuss in the limitations that the absence of PROBAST-based appraisal limits the ability to assess the internal validity of the reported performance metrics. IMPORTANTLY, this does not require re-doing the review, it requires adding interpretive context that is currently missing.

Response: Thank you for raising this important point. We have revisited the six systematic reviews that evaluated the included prediction tools. Of these, two did not use any formal appraisal tool. Among the remaining four, two applied CHARMS, one used PROBAST, and one employed a self-developed assessment tool.

We have now added this information to the Methods section under quality appraisal, as well as to the Results section for clarity. In addition, we have incorporated discussion of risk of bias concerns, including those highlighted in the PROBAST-based assessment by Dewajoren et al., for the two tools that showed the highest performance overall.

The relevant sections now read as follows:

Method 2.4, P10: “*In addition, we extracted whether the systematic reviews applied a specific appraisal tool for prediction models, such as PROBAST (Prediction model Risk Of Bias ASsessment Tool ), to assess risk of bias and applicability of the prediction models [35].”*

*Results section 3.1 p. 13: “Of the six reviews that specifically evaluated risk prediction tools four used a prediction-model specific risk-of bias tool, with two using CHARMS (Checklist for the critical Appraisal and data extraction for systematic Reviews of prediction Modelling Studies) [45,51], one using PROBAST [25] and one developed their own appraisal tool [52].”*

Discussion p. 33: “*However, both appear to have an overall high risk of bias based on the PROBAST assessment [25] and could benefit from further external validation, clearer risk factor definitions and evaluation of their impact on patient prioritisation.”*

2. Exclusive focus on discrimination without consideration of calibration or clinical utility.
The manuscript evaluates tool performance almost entirely through discrimination metrics (AUROC, C-statistic, sensitivity, specificity). While discrimination is important, it is well-established that a model can have acceptable discrimination yet be poorly calibrated (meaning it systematically over- or under-estimates risk) which would render it unsuitable for clinical decision-making. A widely cited tutorial noted that while 63% of published prediction models report discrimination, only 36% report calibration (Huang et al., 2020; Wessler et al., 2016). The authors should add a paragraph in the discussion acknowledging that AUROC alone is insufficient for implementation decisions, that (if) calibration data were absent or unreported for nearly all tools identified, and that this itself is an important finding. The absence of calibration data could be framed as reinforcing the conclusion that no tool is currently implementation-ready, which would actually strengthen the manuscript's argument.

Response: Thank you for raising this point. We agree that the calibration is also an important aspect to evaluate a tool performance and should not be left out. We have now extracted the calibration scores where available for the tools and have added the following sections in our manuscript:

We have now extracted a calibration column in Table 2 and have outlined the definition of Calibration in the method 2.5 (p.11): *“Another key measure of predictive performance is a model’s calibrations, which refers to the agreement between the predicted probability by a model and the actual outcomes. Perfect calibration is indicated by an intercept approaching 0 and a slope approaching 1, whereas a statistically significant Hosmer–Lemeshow test suggests poor model calibration [37].”*

We have also added a statement in the result section (3.3.1, p.20) outlining the findings in regard to the prediction models that reported a calibration*: “Only eight (25%) of the 32 tools reported calibration data. Of these, six reported Hosmer–Lemeshow test results, all of which were statistically non-significant (p values ranging from 0.13 to 0.99), indicating acceptable calibration [62, 65, 82, 84, 88, 91]. Two tools (‘MOAT’ and ‘PRISMOR’) had corrected calibration slopes, which suggested good calibration (0.88 and 0.93 respectively) [75,83].”*

The two previous highlighted tools in the discussion (BADRI and DRP) that had acceptable discrimination as well as external validation results available in addition also both showed good calibration, which further strengthened our conclusion that these two tools are the most promising ones of the tools included in this review. We have now added this in the discussion as well.

Discussion, p.32: *“The identified tools often showed limited or incompletely reported performance in relation to discrimination, calibration, sensitivity, and specificity, all of which are important for clinical implementation. Only six of the 32 tools reported external validation, and only eight provided information on model calibration. In addition, only five tools reported sufficient statistical power based on events per variable (EPV), raising concerns about the validity of the reported AUROC values and, consequently, the predictive performance and clinical applicability of these tools. Of the 32 identified tools, two (i.e. BADRI [62], DRP Risk Score [88,89] showed the highest potential for future clinical use based on their discriminatory ability, calibration and available external validation data.”*

3. Tension between AI-focused conclusions and systematic exclusion of AI literature
The discussion makes a strong case for AI-enabled prediction tools and highlights the AutoMedic project as a translational pathway. However, the eligibility criteria required that systematic reviews report individual risk factors, which led to the exclusion of reviews examining AI/ML and electronic health record-based prediction models (the authors cite Yasrebi-de Kom et al., JAMIA, 2023 [ref 27] and Johns et al., Eur J Hosp Pharm, 2024 [ref 115] as examples). This creates an internal tension: the paper advocates for AI-driven tools while its review design systematically excludes the existing AI literature. This is not necessarily a flaw, and the focus on interpretable risk factors is a defensible design choice, but the implication needs to be made explicit. Currently, the manuscript reads as though the field lacks AI tools, when in fact such tools exist but were excluded by design. The authors should: (a) acknowledge this tension explicitly in the methods (when justifying the eligibility criteria) and in the discussion; and (b) qualify the AI-related conclusions to clarify that they are based on the conventional prediction tool literature and that a separate review of AI/ML-based models would complement this work.

Response: Thank you for raising this and we agree that this point could be made clearer throughout the manuscript. We have made the following revisions throughout the manuscript to address this.

Method section 2.2 (p.9): *“Reviews that only discussed overall prediction models/tools without outlining individual patient or medication characteristics were excluded due to the second aim of this study. Consequently, some reviews that focused on AI, specifically machine learning prediction tools, were not eligible for inclusion because individual contributory risk factors were not detailed [26, 27].”*

Result section 3.3 (p. 19)*:* We have replaced “*identified tools*” with “*included tools*” to more clearly distinguish between existing AI-based prediction models and the scope of tools included in this umbrella review.

Discussion: We have removed wording that could be interpreted as implying that machine-based prediction tools do not already exist. And we have added the following sentence under limitations (p. 37): “*Only systematic reviews that reported specific risk factors were included in this umbrella review. Consequently, the few identified reviews on AI-driven and electronic health record-based prediction models were excluded as the primary studies did not disclose risk factors, potentially due to proprietary reasons [26, 27]. Therefore, a review specifically evaluating AI-based and machine learning prediction models could help identify relevant data-driven approaches that may complement and build upon the findings of the present review. “*

4. Risk factor synthesis limited by unassessed outcome-level overlap
The authors report a global CCA of 3.47%, suggesting low overall overlap across the 18 reviews. However, the CCA was not calculated at the level of individual risk factors or outcomes. The vote-counting approach reporting how many of the 18 reviews identified each risk factor is intuitive but potentially misleading. For example, psycholeptics are reported as the most commonly implicated medication class (n=13 reviews), but it is unclear whether these 13 reviews drew on largely independent primary studies or whether a smaller number of influential primary studies were repeatedly included across reviews. Without factor-level overlap analysis, the frequency counts may overrepresent factors from shared primary studies. The authors should acknowledge in the limitations that the CCA was calculated globally and not at the risk factor level, and that the frequency counts should therefore be interpreted with caution as they may not reflect 13 independent lines of evidence.

Response: We agree that the narrative nature of the risk factor synthesis and the potential overlap between included reviews needed to be more clearly acknowledged. We have now added clarification in the Methods section to explicitly state that the risk factor synthesis was descriptive in nature and based on reporting frequency across reviews (please see our response to Reviewer 2, Comment 4).

In addition, we have included the following statement in the limitations section of the Discussion (p. 37):

*“In addition, the synthesis of risk factors was based on frequency of reporting across systematic reviews rather than quantitative pooling of effect sizes or formal assessment of the consistency and strength of associations across primary studies.”*

Minor Issues

5. Events per variable (EPV) deserves greater emphasis.
The authors note that only 5 of 32 tools had sufficient statistical power based on EPV, 4 were underpowered, and 23 were unclear or not reported. This is mentioned in passing, but is a major threat to the validity of the reported AUROC values, as underpowered models are prone to overfitting and optimistic performance estimates. This finding should be highlighted more prominently in the discussion and limitations, as it further supports the conclusion that most tools are not ready for clinical use.

Response: Thank you for highlighting the importance of events per variable (EPV). We agree that insufficient statistical power represents a major limitation and may contribute to overfitting and overly optimistic performance estimates. To address this point, we have expanded the discussion to emphasise the limited reporting of EPV, external validation, and calibration across the identified tools.

We added the following paragraph to the discussion (p. 32): “Only six of the 32 tools reported external validation, and only eight provided information on model calibration. In addition, only five tools reported sufficient statistical power based on events per variable (EPV), raising concerns about the validity of the reported AUROC values and, consequently, the predictive performance and clinical applicability of these tools.”

6. Heterogeneity across tools warrants a stronger caveat.
Table 2 presents 32 tools side by side, but they predict different outcomes (ADEs, ADRs, MEs, DRPs broadly, readmission, pharmacist intervention), in different populations (adults ≥15y to ≥80y), across different settings, and with variable counts ranging from 3 to 310. Comparing AUROCs across tools that are solving fundamentally different prediction problems risks false equivalence. A brief statement in the discussion or at the beginning of section 3.3 acknowledging this heterogeneity and cautioning against direct comparison would be appropriate.

Response: Thank you for noting this. We have now expanded upon this point in the discussion (p. 33): “*Additionally, substantial heterogeneity existed in terms of predicted outcomes (e.g., ADRs, ADEs, medication errors, DRPs, readmission, or pharmacist intervention), patient populations, clinical settings, and number of predictors included across the tools. As a result, direct comparisons of model performance, including AUROC values, should be interpreted with caution. This heterogeneity also limits the generalisability of findings across hospital settings and patient populations”*

Overall Recommendation

This is a very well-conducted umbrella review addressing a clinically important question. The main revisions needed are additions to the discussion and limitations rather than changes to the underlying methodology. Specifically, the authors should address the PROBAST gap, add calibration/clinical utility context, resolve the AI exclusion tension, and strengthen the risk factor overlap caveat. With these revisions, the manuscript would make a solid contribution to PLOS Digital Health.

Response: Thank you again for your constructive feedback. We have addressed the points raised throughout the manuscript, including clarification of risk of bias considerations (including PROBAST), additional context on calibration and clinical utility, clarification of the scope regarding AI-based tools, and a strengthened discussion of risk factor overlap and related limitations. We hope the revised manuscript now meets your expectations.

Reference:

1.	Deawjaroen K, Sillabutra J, Poolsup N, Stewart D, Suksomboon N. Clinical usefulness of prediction tools to identify adult hospitalized patients at risk of drug-related problems: A systematic review of clinical prediction models and risk assessment tools. Br J Clin Pharmacol. 2022;88(4):1613-29.
