# Supplementary material for: Prediction tools to prioritise hospitalised adult patients at risk of drug related problems: An umbrella review
Source: PLOS Digit Health. 2026 Aug 6;5(8):e0001583. doi: 10.1371/journal.pdig.0001583 (PMC13446687; doi:10.1371/journal.pdig.0001583)
Supplement: Peer review history

# Peer review: Decision Letter 0

**DOI:** 10.1371/journal.pdig.0001583.r001

**Authors:** Laura Sbaffi, Bushra Ali Sherazi

14 Apr 2026

PDIG-D-26-00198

Prediction tools to prioritise hospitalised adult patients at risk of drug related problems: An umbrella review

PLOS Digital Health

Dear Dr. Babu,

Thank you for submitting your manuscript to PLOS Digital Health. After careful consideration, we feel that it has merit but does not fully meet PLOS Digital Health's publication criteria as it currently stands. Therefore, we invite you to submit a revised version of the manuscript that addresses the points raised during the review process.

Please submit your revised manuscript by Jun 13 2026 11:59PM. If you will need more time than this to complete your revisions, please reply to this message or contact the journal office at digitalhealth@plos.org. When you're ready to submit your revision, log on to https://www.editorialmanager.com/pdig/ and select the 'Submissions Needing Revision' folder to locate your manuscript file.

Please include the following items when submitting your revised manuscript:

* A letter that responds to each point raised by the editor and reviewer(s). You should upload this letter as a separate file labeled 'Response to Reviewers'. This file does not need to include responses to any formatting updates and technical items listed in the 'Journal Requirements' section below.

* A marked-up copy of your manuscript that highlights changes made to the original version. You should upload this as a separate file labeled 'Revised Manuscript with Track Changes'.

* An unmarked version of your revised paper without tracked changes. You should upload this as a separate file labeled 'Manuscript'.

If you would like to make changes to your financial disclosure, competing interests statement, or data availability statement, please make these updates within the submission form at the time of resubmission. Guidelines for resubmitting your figure files are available below the reviewer comments at the end of this letter.

We look forward to receiving your revised manuscript.

Kind regards,

Bushra Ali Sherazi

Guest Editor

PLOS Digital Health

Laura Sbaffi

Section Editor

PLOS Digital Health

Leo Anthony Celi

Editor-in-Chief

PLOS Digital Health

orcid.org/0000-0001-6712-6626

**Journal Requirements:**

1. Please upload separate figure files in .tif or .eps format. Also, remove the figures from your manuscript file but keep the legends.

For more information about figure files please see our guidelines:

https://journals.plos.org/digitalhealth/s/figures

https://journals.plos.org/digitalhealth/s/figures#loc-file-requirements

2. We have noticed that you have uploaded Supporting Information files, but you have not included a list of legends. Please add a full list of legends for your Supporting Information files after the references list.

If the reviewer comments include a recommendation to cite specific previously published works, please review and evaluate these publications to determine whether they are relevant and should be cited. There is no requirement to cite these works unless the editor has indicated otherwise.

**Additional Editor Comments (if provided):**

[Note: HTML markup is below. Please do not edit.]

**Reviewers' Comments:**

Reviewer's Responses to Questions

**Comments to the Author**

1. Does this manuscript meet PLOS Digital Health’s publication criteria? Is the manuscript technically sound, and do the data support the conclusions? The manuscript must describe methodologically and ethically rigorous research with conclusions that are appropriately drawn based on the data presented.

Reviewer #1: Yes

Reviewer #2: Partly

Reviewer #3: Partly

**********

2. Has the statistical analysis been performed appropriately and rigorously?

Reviewer #1: Yes

Reviewer #2: No

Reviewer #3: Yes

**********

3. Have the authors made all data underlying the findings in their manuscript fully available (please refer to the Data Availability Statement at the start of the manuscript PDF file)?

The PLOS Data policy requires authors to make all data underlying the findings described in their manuscript fully available without restriction, with rare exception. The data should be provided as part of the manuscript or its supporting information, or deposited to a public repository. For example, in addition to summary statistics, the data points behind means, medians and variance measures should be available. If there are restrictions on publicly sharing data—e.g. participant privacy or use of data from a third party—those must be specified.

Reviewer #1: Yes

Reviewer #2: Yes

Reviewer #3: Yes

**********

4. Is the manuscript presented in an intelligible fashion and written in standard English?

PLOS Digital Health does not copyedit accepted manuscripts, so the language in submitted articles must be clear, correct, and unambiguous. Any typographical or grammatical errors should be corrected at revision, so please note any specific errors here.

Reviewer #1: Yes

Reviewer #2: Yes

Reviewer #3: Yes

**********

5. Review Comments to the Author

Please use the space provided to explain your answers to the questions above. You may also include additional comments for the author, including concerns about dual publication, research ethics, or publication ethics. (Please upload your review as an attachment if it exceeds 20,000 characters)

**Reviewer #1:**  1. There is a lot of variation in how reviews define "acceptable" and "good" discrimination. This inconsistency can affect how the 32 tools are assessed.

2. Only two tools showed good potential for clinical use, but the manuscript does not explain clearly which populations the validation worked for and which it did not.

3. Although "reduced renal function" is an important factor, the reviews often do not provide a clear definition, such as specific eGFR thresholds. This lack of clarity makes it harder to create automated alerts.

4. The overall clinical classification accuracy (CCA) is low at 3.47%, but there is significant overlap between the findings of different reviews. This suggests that some results may be over-represented.

5. In the Introduction, after discussing automated prioritization tools. It is suggested to refer from the paper: “Analyzing cybersecurity risks and threats in IT infrastructure based on the NIST framework “. This reference is important for discussing the security and risk management needed when implementing "automated, AI-enabled tools" in hospitals. It supports the manuscript by providing a basis for identifying weaknesses in the digital infrastructure that will support these drug-related problem (DRP) prediction tools.

6. The authors need to clarify the area under the receiver operating characteristic curve (AUROC) thresholds used. While a range of 0.70 to 0.80 is described as "acceptable," the authors should explain why tools below this threshold are still included in clinical summaries.

7. A clearer breakdown is needed to explain why current tools are not suitable for regular clinical use. Are the problems due to data silos, a lack of real-time integration with the electronic medical record (EMR), or low sensitivity?

8. The "Author Summary" and "Introduction" should be expanded to better connect existing risk factors with the creation of AI-enabled automated tools.

9. Consistency in terminology is important. For example, "ADR," "ADE," and "DRP" are used interchangeably. A table of definitions in the Introduction would enhance understanding.

**Reviewer #2:**  The manuscript addresses an important and clinically relevant topic, and the authors have applied an appropriate methodological framework, including adherence to PRISMA guidelines, PROSPERO registration, and the use of Joanna Briggs Institute appraisal tools. The literature search appears comprehensive, and the inclusion of a large number of systematic reviews and primary studies provides a valuable overview of the current evidence base.

However, several issues limit the strength and clarity of the manuscript. First, the scope of the study is overly broad. The manuscript attempts to simultaneously identify prediction tools, evaluate their performance, and summarise risk factors. As a result, none of these components are explored in sufficient depth. A clearer prioritisation of objectives would improve the overall coherence and analytical strength of the paper.

Second, the conclusions are somewhat stronger than what the data can support. In particular, the statement that no tools are suitable for routine clinical practice is not adequately justified, as the manuscript does not define explicit criteria for clinical suitability nor systematically assess clinical utility. A more cautious and clearly defined interpretation would be appropriate.

Third, the evaluation of prediction tools is limited primarily to discrimination metrics such as AUROC, without consideration of other important aspects of model performance, including calibration and clinical usefulness. This restricts the ability to draw meaningful conclusions about real-world applicability.

Fourth, the synthesis of risk factors is largely descriptive, based on frequency of reporting across reviews, without consideration of effect size, consistency, or methodological quality. This approach should be interpreted with caution and more clearly framed as a narrative summary rather than a quantitative synthesis.

Finally, the discussion of artificial intelligence is speculative and not directly supported by the data presented in the review. This section should be toned down or more clearly presented as a future perspective.

In summary, while the manuscript has clear strengths and addresses an important topic, substantial revision is required to improve focus, methodological depth, and alignment between results and conclusions.

**Reviewer #3:**  This umbrella review synthesizes 18 systematic reviews encompassing 171 unique primary studies and 32 risk prediction tools for drug-related problems (DRPs) in hospitalized adults. The topic is timely and practically important: pharmacists need evidence-based tools for patient prioritization, and this review provides a useful landscape analysis. The umbrella review design is well-suited to the mature state of this literature, and the authors followed JBI methodology with a registered protocol on PROSPERO. The overlap analysis, quality appraisal, and detailed tool-level extraction in Table 2 are strengths.

However, some issues require attention before the manuscript is suitable for publication. I have organized these into major issues (which require revision) and minor issues (which could be addressed through additional limitation language or brief additions). I note that my observations are about strengthening an already useful contribution, not about fundamental flaws in the study design.

Major Issues

1. Absence of prediction model-specific risk-of-bias assessment (PROBAST).

The authors used the JBI critical appraisal checklist for systematic reviews to assess the quality of the included reviews. This is appropriate for appraising the reviews as reviews. However, the primary subject matter of this umbrella review is the performance of prediction models, and the manuscript does not discuss whether the included systematic reviews themselves used any prediction model-specific risk-of-bias tool to appraise their primary studies. PROBAST (Prediction model Risk Of Bias ASsessment Tool) is the consensus standard for assessing risk of bias and applicability in prediction model studies. This is directly relevant to interpreting the AUROC values in Table 2. The authors should: (a) report whether any of the six systematic reviews that evaluated risk prediction tools used PROBAST or an equivalent prediction model-specific tool; and (b) discuss in the limitations that the absence of PROBAST-based appraisal limits the ability to assess the internal validity of the reported performance metrics. IMPORTANTLY, this does not require re-doing the review, it requires adding interpretive context that is currently missing.

2. Exclusive focus on discrimination without consideration of calibration or clinical utility.

The manuscript evaluates tool performance almost entirely through discrimination metrics (AUROC, C-statistic, sensitivity, specificity). While discrimination is important, it is well-established that a model can have acceptable discrimination yet be poorly calibrated (meaning it systematically over- or under-estimates risk) which would render it unsuitable for clinical decision-making. A widely cited tutorial noted that while 63% of published prediction models report discrimination, only 36% report calibration (Huang et al., 2020; Wessler et al., 2016). The authors should add a paragraph in the discussion acknowledging that AUROC alone is insufficient for implementation decisions, that (if) calibration data were absent or unreported for nearly all tools identified, and that this itself is an important finding. The absence of calibration data could be framed as reinforcing the conclusion that no tool is currently implementation-ready, which would actually strengthen the manuscript's argument.

3. Tension between AI-focused conclusions and systematic exclusion of AI literature

The discussion makes a strong case for AI-enabled prediction tools and highlights the AutoMedic project as a translational pathway. However, the eligibility criteria required that systematic reviews report individual risk factors, which led to the exclusion of reviews examining AI/ML and electronic health record-based prediction models (the authors cite Yasrebi-de Kom et al., JAMIA, 2023 [ref 27] and Johns et al., Eur J Hosp Pharm, 2024 [ref 115] as examples). This creates an internal tension: the paper advocates for AI-driven tools while its review design systematically excludes the existing AI literature. This is not necessarily a flaw, and the focus on interpretable risk factors is a defensible design choice, but the implication needs to be made explicit. Currently, the manuscript reads as though the field lacks AI tools, when in fact such tools exist but were excluded by design. The authors should: (a) acknowledge this tension explicitly in the methods (when justifying the eligibility criteria) and in the discussion; and (b) qualify the AI-related conclusions to clarify that they are based on the conventional prediction tool literature and that a separate review of AI/ML-based models would complement this work.

4. Risk factor synthesis limited by unassessed outcome-level overlap

The authors report a global CCA of 3.47%, suggesting low overall overlap across the 18 reviews. However, the CCA was not calculated at the level of individual risk factors or outcomes. The vote-counting approach reporting how many of the 18 reviews identified each risk factor is intuitive but potentially misleading. For example, psycholeptics are reported as the most commonly implicated medication class (n=13 reviews), but it is unclear whether these 13 reviews drew on largely independent primary studies or whether a smaller number of influential primary studies were repeatedly included across reviews. Without factor-level overlap analysis, the frequency counts may overrepresent factors from shared primary studies. The authors should acknowledge in the limitations that the CCA was calculated globally and not at the risk factor level, and that the frequency counts should therefore be interpreted with caution as they may not reflect 13 independent lines of evidence.

Minor Issues

5. Events per variable (EPV) deserves greater emphasis.

The authors note that only 5 of 32 tools had sufficient statistical power based on EPV, 4 were underpowered, and 23 were unclear or not reported. This is mentioned in passing, but is a major threat to the validity of the reported AUROC values, as underpowered models are prone to overfitting and optimistic performance estimates. This finding should be highlighted more prominently in the discussion and limitations, as it further supports the conclusion that most tools are not ready for clinical use.

6. Heterogeneity across tools warrants a stronger caveat.

Table 2 presents 32 tools side by side, but they predict different outcomes (ADEs, ADRs, MEs, DRPs broadly, readmission, pharmacist intervention), in different populations (adults ≥15y to ≥80y), across different settings, and with variable counts ranging from 3 to 310. Comparing AUROCs across tools that are solving fundamentally different prediction problems risks false equivalence. A brief statement in the discussion or at the beginning of section 3.3 acknowledging this heterogeneity and cautioning against direct comparison would be appropriate.

Overall Recommendation

This is a very well-conducted umbrella review addressing a clinically important question. The main revisions needed are additions to the discussion and limitations rather than changes to the underlying methodology. Specifically, the authors should address the PROBAST gap, add calibration/clinical utility context, resolve the AI exclusion tension, and strengthen the risk factor overlap caveat. With these revisions, the manuscript would make a solid contribution to PLOS Digital Health.

**********

6. PLOS authors have the option to publish the peer review history of their article (what does this mean?). If published, this will include your full peer review and any attached files.

**Do you want your identity to be public for this peer review?**  If you choose “no”, your identity will remain anonymous but your review may still be made public.

For information about this choice, including consent withdrawal, please see our Privacy Policy.

Reviewer #1: No

Reviewer #2: No

Reviewer #3: No

**********

[NOTE: If reviewer comments were submitted as an attachment file, they will be attached to this email and accessible via the submission site. Please log into your account, locate the manuscript record, and check for the action link "View Attachments". If this link does not appear, there are no attachment files.]

**Figure resubmission:**

While revising your submission, we strongly recommend that you use PLOS’s NAAS tool (https://ngplosjournals.pagemajik.ai/artanalysis) to test your figure files. NAAS can convert your figure files to the TIFF file type and meet basic requirements (such as print size, resolution), or provide you with a report on issues that do not meet our requirements and that NAAS cannot fix.

After uploading your figures to PLOS’s NAAS tool - https://ngplosjournals.pagemajik.ai/artanalysis, NAAS will process the files provided and display the results in the "Uploaded Files" section of the page as the processing is complete. If the uploaded figures meet our requirements (or NAAS is able to fix the files to meet our requirements), the figure will be marked as "fixed" above. If NAAS is unable to fix the files, a red "failed" label will appear above. When NAAS has confirmed that the figure files meet our requirements, please download the file via the download option, and include these NAAS processed figure files when submitting your revised manuscript.

**Reproducibility:**

To enhance the reproducibility of your results, we recommend that authors of applicable studies deposit laboratory protocols in protocols.io, where a protocol can be assigned its own identifier (DOI) such that it can be cited independently in the future. Additionally, PLOS ONE offers an option to publish peer-reviewed clinical study protocols. Read more information on sharing protocols at https://plos.org/protocols?utm_medium=editorial-email&utm_source=authorletters&utm_campaign=protocols

# Author comment: Author response to Decision Letter 1

**DOI:** 10.1371/journal.pdig.0001583.r002

11 Jun 2026

Attachment Submitted filename: Response to reviewers.docx

# Editor report: Decision Letter 1

**DOI:** 10.1371/journal.pdig.0001583.r003

**Authors:** Laura Sbaffi, Bushra Ali Sherazi

30 Jun 2026

Prediction tools to prioritise hospitalised adult patients at risk of drug related problems: An umbrella review

PDIG-D-26-00198R1

Dear Dr Babu,

We are pleased to inform you that your manuscript 'Prediction tools to prioritise hospitalised adult patients at risk of drug related problems: An umbrella review' has been provisionally accepted for publication in PLOS Digital Health.

Before your manuscript can be formally accepted you will need to complete some formatting changes, which you will receive in a follow-up email from a member of our team.

Please note that your manuscript will not be scheduled for publication until you have made the required changes, so a swift response is appreciated.

IMPORTANT: The editorial review process is now complete. PLOS will only permit corrections to spelling, formatting or significant scientific errors from this point onwards. Requests for major changes, or any which affect the scientific understanding of your work, will cause delays to the publication date of your manuscript.

If your institution or institutions have a press office, please notify them about your upcoming paper to help maximize its impact. If they'll be preparing press materials, please inform our press team as soon as possible -- no later than 48 hours after receiving the formal acceptance. Your manuscript will remain under strict press embargo until 2 pm Eastern Time on the date of publication. For more information, please contact digitalhealth@plos.org.

Thank you again for supporting Open Access publishing; we are looking forward to publishing your work in PLOS Digital Health.

Best regards,

Bushra Ali Sherazi

Academic Editor

PLOS Digital Health

***********************************************************

**Additional Editor Comments (if provided):**

**Reviewer Comments (if any, and for reference):**
